# Supplementary material for: Molecularly Guided Drug Repurposing for Cholangiocarcinoma: An Integrative Bioinformatic Approach
Source: Genes (Basel). 2022 Jan 29;13(2):271. doi: 10.3390/genes13020271 (PMC8871819; doi:10.3390/genes13020271)
Supplement: Supplementary file 1 [file genes-13-00271-s001.zip › Supplementary figures_R1_NEW.pdf]

a

b

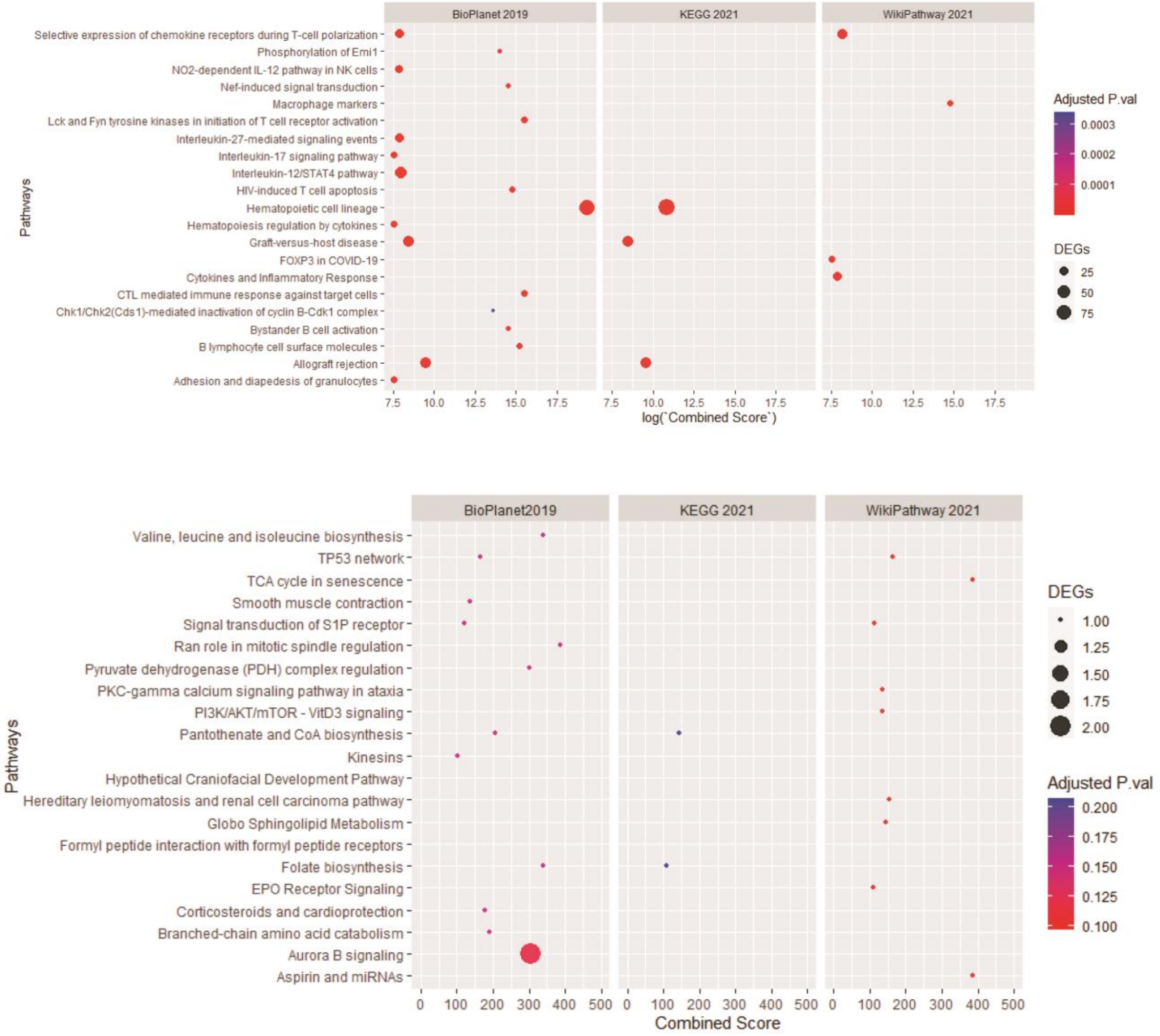

Supplementary Figure S1 – (a) Pathway Enrichment Analysis of the full Immune-Oncogenic Signature from 'Immune Landscape of Cancer' against BioPlanet 2019, KEGG 2021, and WikiPathways 2021. (b) Pathway Enrichment Analysis of filtered clinically relevant immune signature for CCA against BioPlanet 2019, KEGG 2021, and WikiPathways 2021 databases.

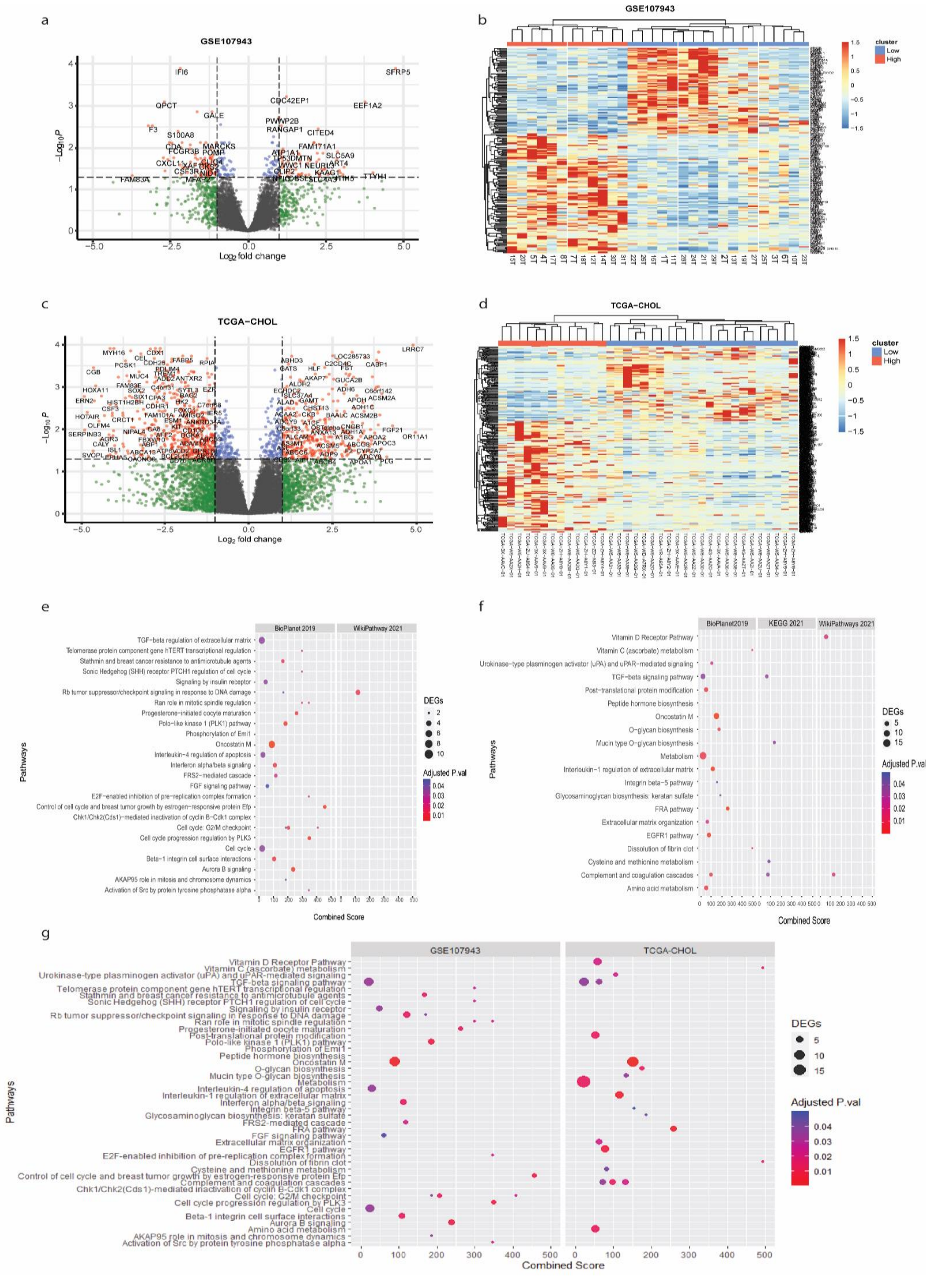

Supplementary Figure S2 - Differential gene expression and pathway enrichment analysis of CCA tumor transcriptomes. (a) Volcano Plot between the low- vs. high-expression groups in the GSE107943 cohort. Red dots indicate 150 significant DEGs with 2x fold-changes and adjusted p-value<0.05. (b) Heatmap of 150 significant DEGs derived from the Volcano plot in the GSE107943 cohort. (c) Volcano Plot between the low- vs. high-expression groups in TCGA-CHOL. Red dots indicate 888 significant DEGs with 2x fold-changes and adjusted p-value<0.05. (d) Heatmap of 888 significant DEGs derived from the Volcano plot in TCGA-CHOL. (e) Pathway enrichment analysis of 150 significant DEGs against BioPlanet2019, KEGG 2021, and Wiki Pathways 2021 in GSE107943 cohort. (f) Pathway enrichment analysis of 888 significant DEGs against BioPlanet2019, KEGG 2021, and Wiki Pathways 2021 in TCGA-CHOL cohort. (g) Significantly enriched pathways from both datasets.

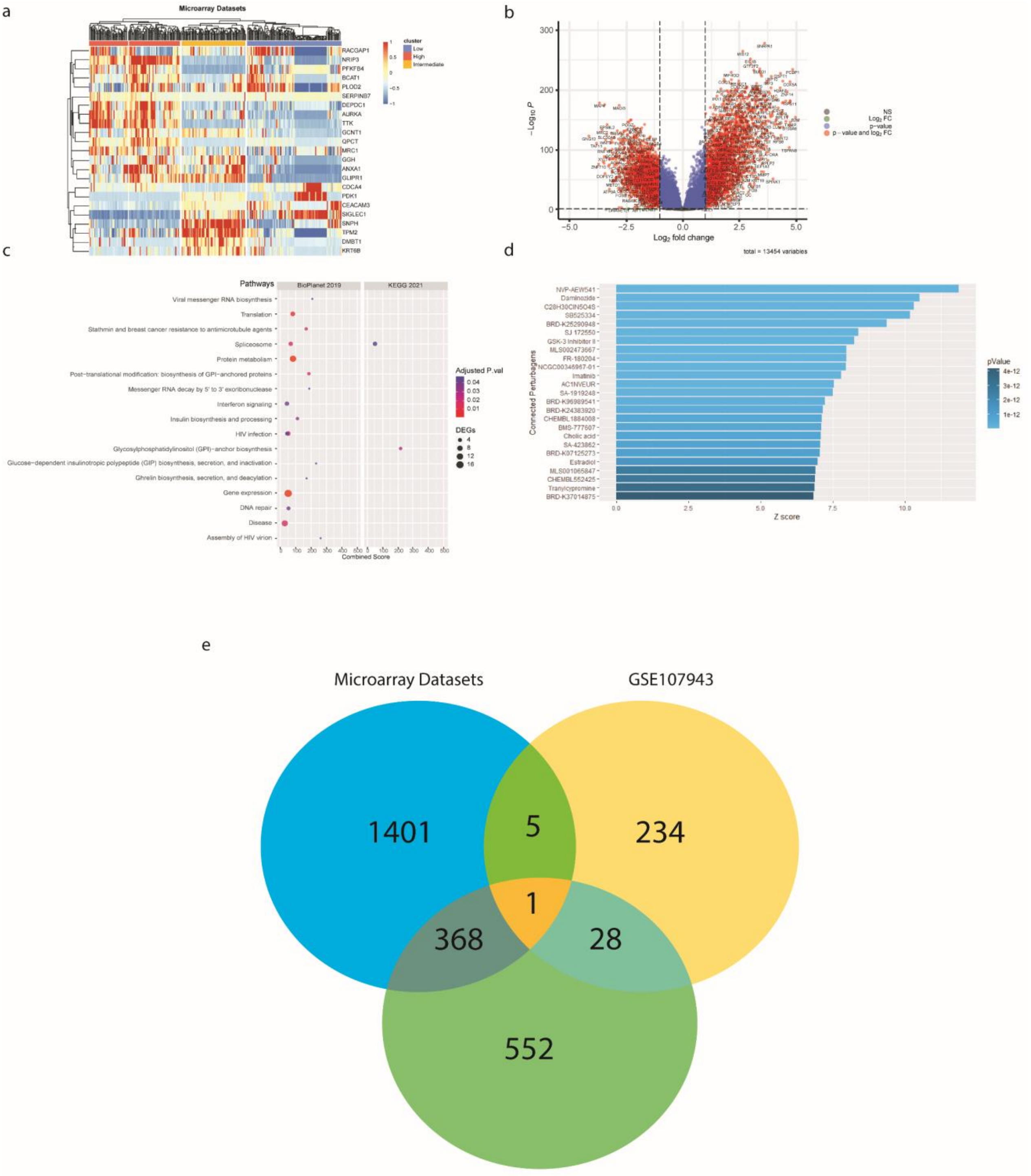

Supplementary Figure S3 - (a) Unsupervised Hierarchical clustering of CCA samples from 10 Microarray datasets against the CCA immune signature. (b) Differential Gene Expression analysis of low vs. high expressing CCA samples from the Microarray cohorts. (c) Pathway enrichment analysis of the DEGs against BioPlanet 2019, and KEGG 2021. (d) Top 25 connected perturbagens positively correlated to transcriptional changes between the low- vs. high-expression groups in the GSE107943 cohort. (e) Venn Diagram depicting the common perturbagens identified in each analysis.
