# Supplementary material for: Molecularly Guided Drug Repurposing for Cholangiocarcinoma: An Integrative Bioinformatic Approach
Source: Genes (Basel). 2022 Jan 29;13(2):271. doi: 10.3390/genes13020271 (PMC8871819; doi:10.3390/genes13020271)
Supplement: Supplementary file 1 [file genes-13-00271-s001.zip › Suppl_Rscript_datasets.zip!/Suppl_Rscript_datasets/CCAImmuneSig_final.pdf]

R Script for CCA Immune Signature Analysis

Code

Simran Venkatraman and Somchai Chutipongtanate

This is an R Markdown (http://rmarkdown.rstudio.com) Notebook. When you execute code within the notebook, the results appear beneath the code.

Try executing this chunk by clicking the *Run* button within the chunk or by placing your cursor inside it and pressing *Ctrl+Shift+Enter*.

First, install the package dependencies required for this analysis. Required package libraries will be loaded in their respective steps.

Hide

```
#Install Package Dependencies
install.packages(c("readr", "survival", "ranger", "dplyr", "ggplot2", "ggfortify",
"survminer","ggrepel", "tidyr", "tibble", "pheatmap", "stringr"))
BiocManager::install(c("edgeR", "EnhancedVolcano", "DESeq2", "limma"))
```

Hide

```
#Load Libraries
library(readr)
library(survival)
library(ranger)
library(ggplot2)
library(dplyr)
library(ggfortify)
library(survminer)
library(ggrepel)
library(tidyr)
library(tibble)
library(limma)
library(edgeR)
library(stringr)
```

Download the Input datasets onto your desktop directory to load into R (available in Supplementary Information).

For the first analysis (Univariate Cox-Proportional Hazards Model and Kaplain-Meier analysis of genes) we will use “GSEsurvgenes.csv” and “TCGAsurvgenes.csv”. These are survival data for Overall Survival and Progression Free Survival from the respective cohorts: GSE107943 and TCGA-CHOL, appended with gene expression information of the 3017 genes from the Immune Gene Signature (Table S1)

Hide

```
#Data Loading
setwd("~/Desktop/Datasets/")
GSEsurvgenes <- read_csv("~/Desktop/Datasets/GSEsurvgenes.csv")
TCGAsurvgenes <- read_csv("~/Desktop/Datasets/TCGAsurvgenes.csv")
colnames(GSEsurvgenes) <- gsub(x = colnames(GSEsurvgenes), pattern = "\\-", replac
ement = "")
colnames(TCGAsurvgenes) <- gsub(x = colnames(TCGAsurvgenes), pattern = "\\-", repl
acement = "")
```

Hide

```
#Preparing the function
covariatesGSE <- colnames(GSEsurvgenes[,6:2598])
covariatesTCGA <- colnames(TCGAsurvgenes[,6:2659])
univ_formulas1 <- sapply(covariatesGSE, function(x) as.formula(paste('Surv(OS,Deat
h)~', x)))
univ_formulas2 <- sapply(covariatesTCGA, function(x) as.formula(paste('Surv(OS,Dea
th)~', x)))
univ_models1 <- lapply(univ_formulas1, function(x){coxph(x, data = GSEsurvgenes)})
univ_models2 <- lapply(univ_formulas2, function(x){coxph(x, data = TCGAsurvgenes)}
)
# Applying the function and extracting the results
univ_GSEresults <- lapply(univ_models1,
                       function(x){
                         x <- summary(x)
                         p.value<-signif(x$wald["pvalue"], digits=2)
                         wald.test<-signif(x$wald["test"], digits=2)
                         beta<-signif(x$coef[1], digits=2);#coeficient beta
                         HR <-signif(x$coef[2], digits=2);#exp(beta)
                         HR.confint.lower <- signif(x$conf.int[,"lower .95"], 2)
                         HR.confint.upper <- signif(x$conf.int[,"upper .95"],2)
                         HR <- paste0(HR, " (",
                                      HR.confint.lower, "-", HR.confint.upper, ")"
)
                         res<-c(beta, HR, wald.test, p.value)
                         names(res)<-c("beta", "HR (95% CI for HR)", "wald.test",
                                       "p.value")
                         return(res)
                         #return(exp(cbind(coef(x),confint(x))))
                       })
resGSE <- t(as.data.frame(univ_GSEresults, check.names = FALSE))
resGSE <- as.data.frame(resGSE)

univ_TCGAresults <- lapply(univ_models2,
                          function(x){
                            x <- summary(x)
                            p.value<-signif(x$wald["pvalue"], digits=2)
                            wald.test<-signif(x$wald["test"], digits=2)
                            beta<-signif(x$coef[1], digits=2);#coeficient beta
                            HR <-signif(x$coef[2], digits=2);#exp(beta)
                            HR.confint.lower <- signif(x$conf.int[,"lower .95"], 2
)
                            HR.confint.upper <- signif(x$conf.int[,"upper .95"],2)
```

```
                            HR <- paste0(HR, " (",
                                         HR.confint.lower, "-", HR.confint.upper,
")")
                            res<-c(beta, HR, wald.test, p.value)
                            names(res)<-c("beta", "HR (95% CI for HR)", "wald.test
",
                                          "p.value")
                            return(res)
                            #return(exp(cbind(coef(x),confint(x))))
                          })
resTCGA <- t(as.data.frame(univ_TCGAresults, check.names = FALSE))
resTCGA <- as.data.frame(resTCGA)

#Write the output files
write.csv(resGSE, "~/Desktop/Datasets/TableS2.csv")
write.csv(resTCGA, "~/Desktop/Datasets/TableS3.csv")
```

Hide

```
#Draw a scatter plot depicting significant and insignificant genes
resTCGA$color <- "Insignificant"
resGSE$color <- "Insignificant"
resGSE <- separate(resGSE, `HR (95% CI for HR)`, into = c("HR", "CI"), sep = " (?=
[^ ]+$)")
resTCGA <- separate(resTCGA, `HR (95% CI for HR)`, into = c("HR", "CI"), sep = " (
?=[^ ]+$)")
resGSE[,c(1,2,4,5)] <- sapply(resGSE[,c(1,2,4,5)], as.numeric)
resTCGA[,c(1,2,4,5)] <- sapply(resTCGA[,c(1,2,4,5)], as.numeric)
resTCGA$color[resTCGA$HR > 1 & resTCGA$p.value < 0.05] <- "Significant"
resGSE$color[resGSE$HR > 1 & resGSE$p.value < 0.05] <- "Significant"
```

Hide

```
#GSE Scatter Plot
ggplot(data=resGSE, aes(x=HR, y=-log10(p.value), col = color, label= rownames(resG
SE))) +
geom_point() +
theme_minimal() +
geom_text_repel() +
scale_color_manual(values=c("black", "blue"))+xlim(0, 5) +  geom_vline(xintercept=
1, col="red") + geom_hline(yintercept = 1.3, col = "red")
```

```
Warning: Removed 80 rows containing missing values (geom_point).
Warning: Removed 80 rows containing missing values (geom_text_repel).
Warning: ggrepel: 2482 unlabeled data points (too many overlaps). Consider increas
ing max.overlaps
```

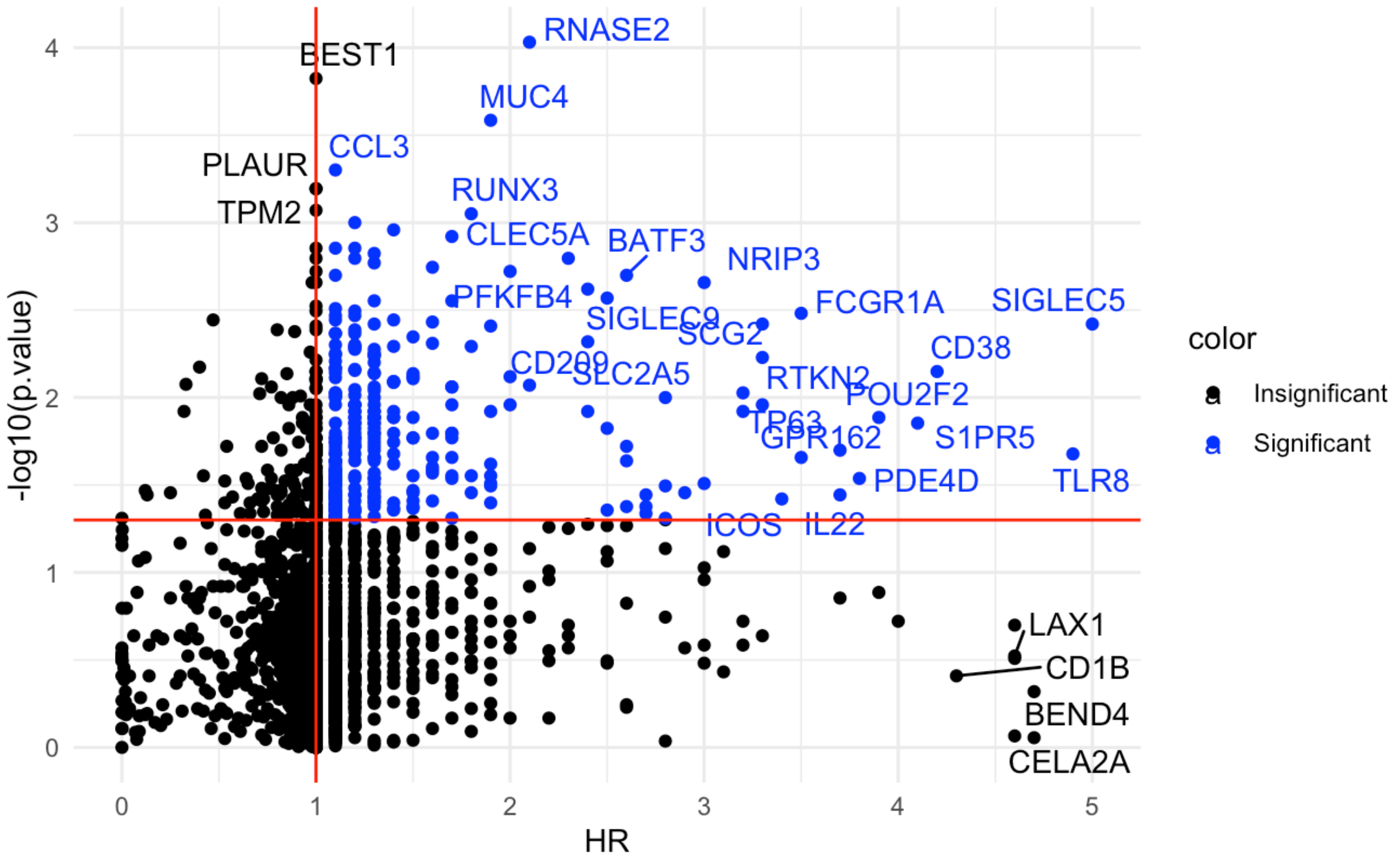

Hide

```
#TCGA Scatter Plot
ggplot(data=resTCGA, aes(x=HR, y=-log10(p.value), col = color, label= rownames(res
TCGA))) +
geom_point() +
theme_minimal() +
geom_text_repel() +
scale_color_manual(values=c("black", "blue"))+xlim(0, 3) +  geom_vline(xintercept=
1, col="red") + geom_hline(yintercept = 1.3, col = "red")
```

```
Warning: Removed 5 rows containing missing values (geom_point).
Warning: Removed 5 rows containing missing values (geom_text_repel).
Warning: ggrepel: 2641 unlabeled data points (too many overlaps). Consider increas
ing max.overlaps
```

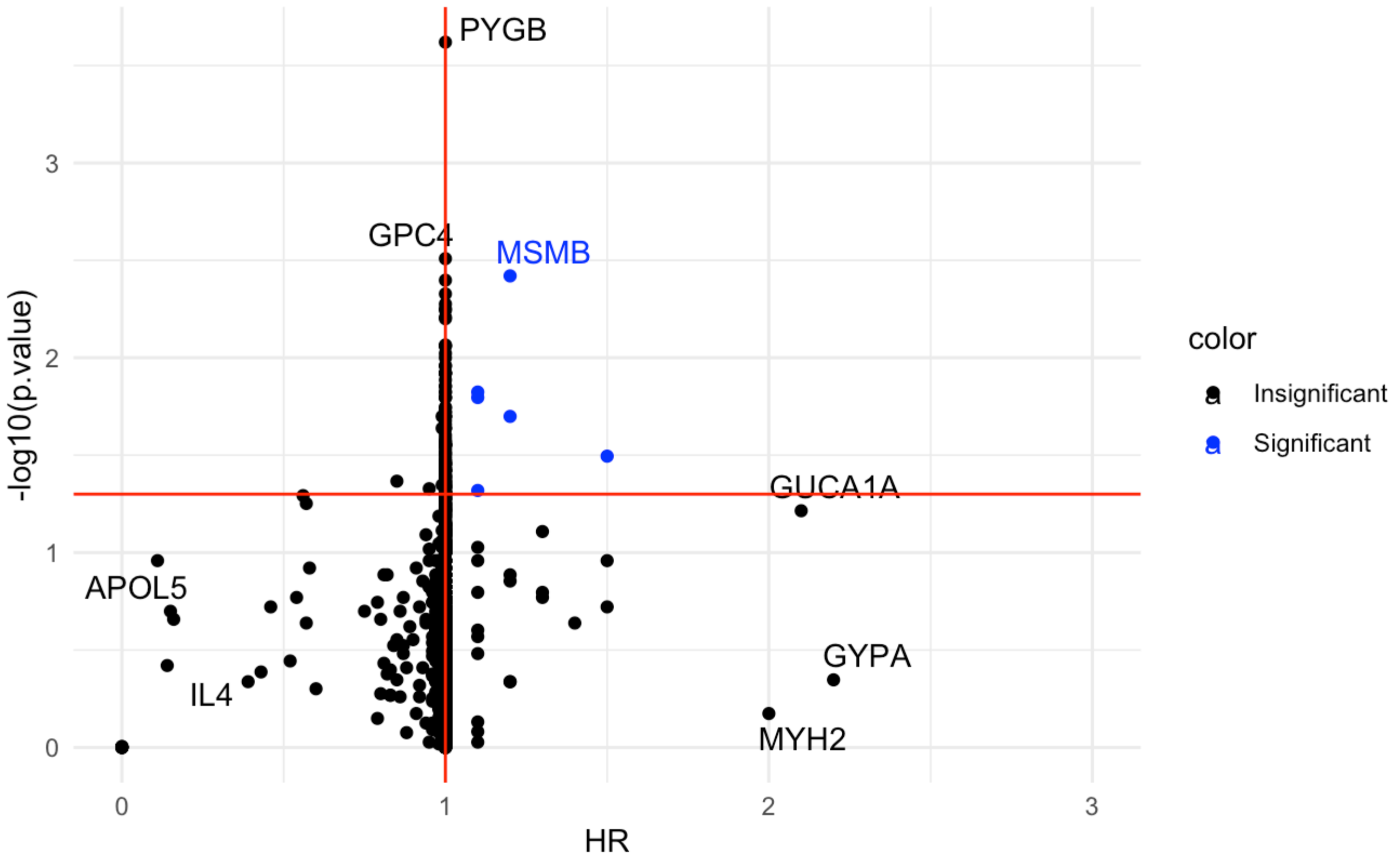

Hide

```
NA
```

Now that we have identified the clinically relevant genes in each cohort, we have enlisted the mutual significant entities in Table S4. We will now use this gene list as the CCA Immune Signature to stratify samples. We will first load the gene expression datasets: “GSEds.csv” and “data_RNA_Seq_v2_expression_median.txt”

Hide

```
#Load Libraries
library(dplyr)
library(pheatmap)
library(tidyr)
library(tibble)

#Load Data
Microarrayds <- read_csv("~/Desktop/Datasets/Microarrayds.csv")
GSEds <- read_csv("~/Desktop/Datasets/GSEds.csv")
GSEds <- GSEds[,-1]
TCGAds <- read_tsv("~/Desktop/Datasets/data_RNA_Seq_v2_expression_median.txt")
TCGAds <- TCGAds[30:20531,-2]
TCGAds <- TCGAds %>% group_by(Hugo_Symbol) %>% summarize_all(mean)

# 26 mortality-associated immune-related genes
genelist <- c("GGH", "ANXA1", "GLIPR1", "PDK1", "TPM2", "SNPH", "DMBT1", "SIGLEC1"
, "KRT6B", "PFKFB4", "CENPW", "RACGAP1", "CDCA4", "BCAT1", "QPCT", "VTA1", "TTK",
"PLOD2", "MRC1", "GCNT1", "SERPINB7", "TP63", "AURKA", "CEACAM3", "NRIP3", "DEPDC1
")
```

```
#Filter datasets for CCA Immune Signature
GSEimmune <- filter(GSEds, GSEds$Genes %in% genelist)
GSEimmune <- column_to_rownames(GSEimmune, "Genes")
#GSEimmune <- GSEimmune[,-1]
TCGAimmune <- filter(TCGAds, TCGAds$Hugo_Symbol %in% genelist)
TCGAimmune <- column_to_rownames(TCGAimmune, "Hugo_Symbol")
Microarrayimmune <- filter(Microarrayds, Microarrayds$GeneID %in% genelist)
Microarrayimmune <- column_to_rownames(Microarrayimmune, "GeneID")

#Hierarchical Clustering
x= pheatmap(GSEimmune, scale = "row", border_color = NA,
         breaks = seq(-1.5, 1.5, length.out = 101),
         clustering_method = "complete",
         clustering_distance_cols = "canberra",
         cutree_cols = 2)
y = pheatmap(TCGAimmune, scale = "row", border_color = NA,
         breaks = seq(-1.5, 1.5, length.out = 101),
         clustering_method = "complete",
         clustering_distance_cols = "canberra",
         cutree_cols = 4)
z = pheatmap(Microarrayimmune, scale = "row", clustering_method = "complete", clus
tering_distance_cols = "canberra", breaks = seq(-1, 1, length.out = 101), show_col
names = F, cutree_cols = 4)

#Identify Cluster IDs
indGSE <- cutree(x$tree_col, k = 2)
indTCGA <- cutree(y$tree_col, k = 4)
indMA <- cutree(z$tree_col, k = 4)
indGSE <- as.data.frame(indGSE)
indTCGA <- as.data.frame(indTCGA)
indMA <- as.data.frame(indMA)
indGSE$indGSE <- sapply(indGSE$indGSE, as.factor)
indTCGA$indTCGA <- sapply(indTCGA$indTCGA, as.factor)
indMA$indMA <- sapply(indMA$indMA, as.factor)
indMA$anno <- "High"
indMA$anno[indMA$indMA == 1] <- "Intermediate"
indMA$anno[indMA$indMA == 4] <- "Low"
write.csv(indGSE, "~/Desktop/Datasets/GSEhclustidentifiers.csv")
write.csv(indTCGA, "~/Desktop/Datasets/TCGAhclustidentifiers.csv")
write.csv(indMA, "~/Desktop/Datasets/MAhclustidentifiers.csv")
```

Hide

```
#Draw Heatmap for GSEimmune
pheatmap(GSEimmune, scale = "row", border_color = NA,
             breaks = seq(-1.5, 1.5, length.out = 101),
             clustering_method = "complete",
             clustering_distance_cols = "canberra",
             cutree_cols = 2, annotation = indGSE, main ="GSE107943")
```

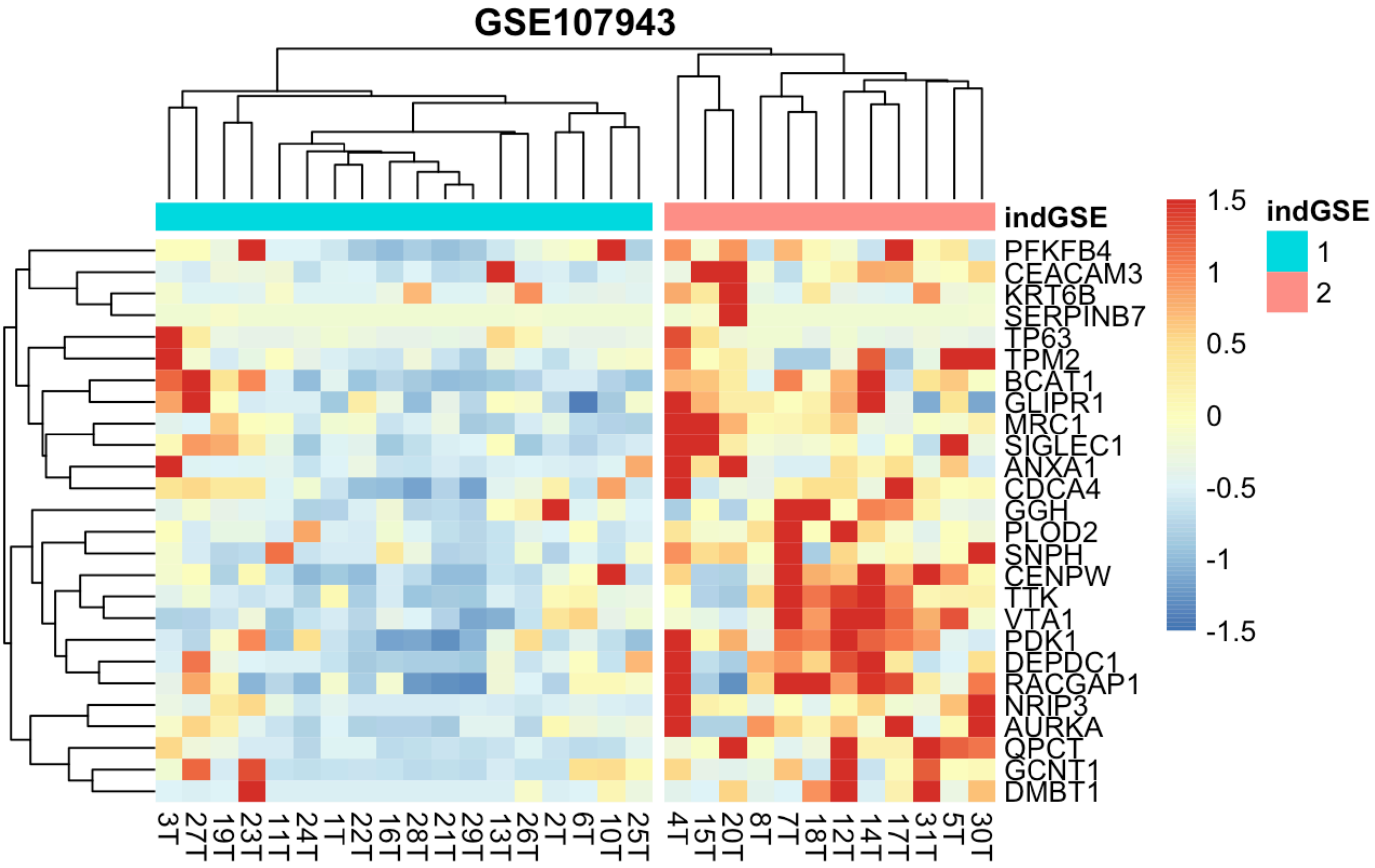

Hide

```
#Draw Heatmap for TCGAimmune
pheatmap(TCGAimmune, scale = "row", border_color = NA,
              breaks = seq(-1.5, 1.5, length.out = 101),
              clustering_method = "complete",
              clustering_distance_cols = "canberra",
              clustering_distance_rows = "manhattan",
              cutree_cols = 4, annotation = indTCGA, main = "TCGA-CHOL")
```

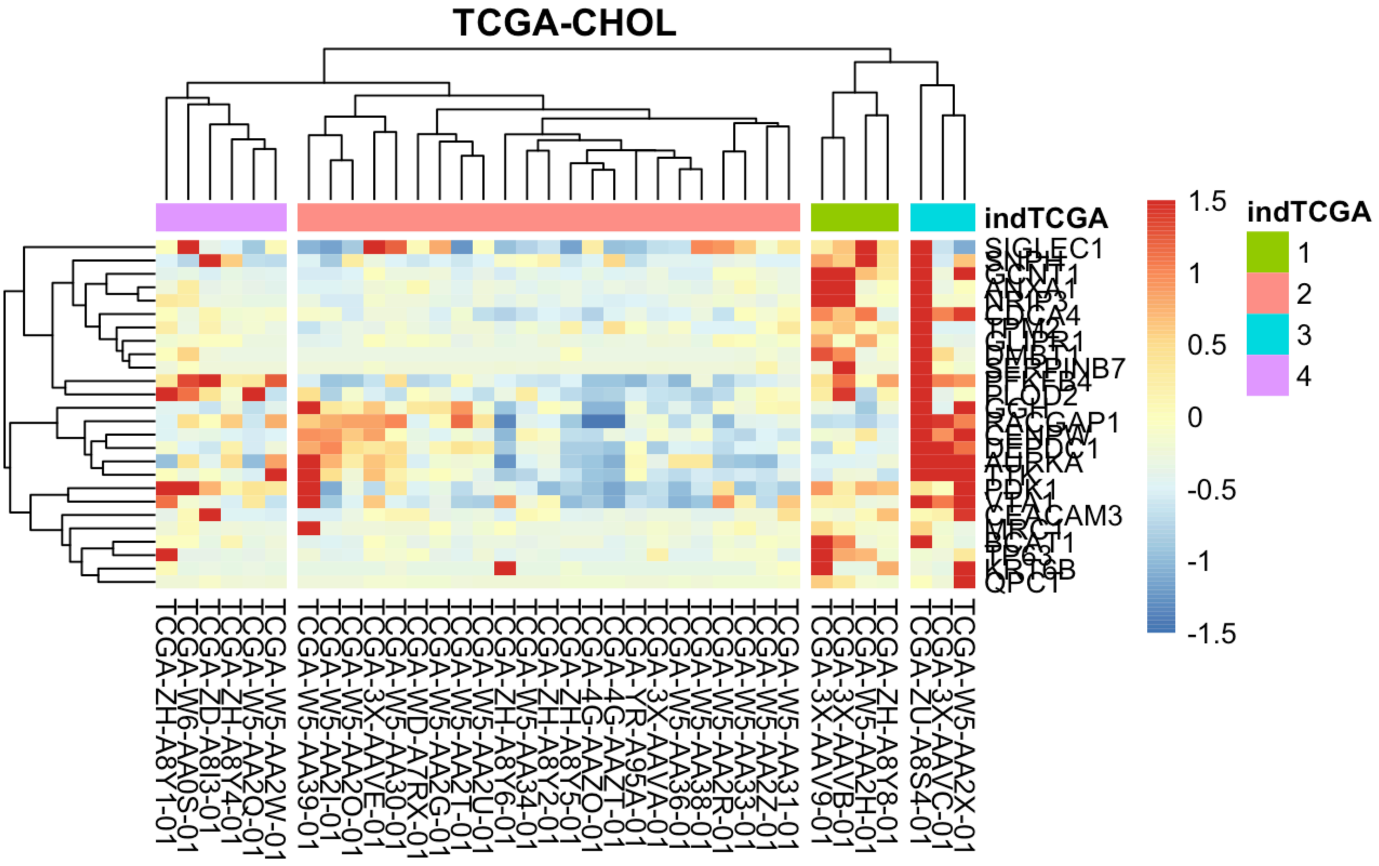

Hide

```
#Draw Heatmap for Microarrayimmune
pheatmap(Microarrayimmune, scale = "row",
         clustering_method = "complete",
         clustering_distance_cols = "canberra",
         breaks = seq(-1, 1, length.out = 101),
         show_colnames = F, cutree_cols = 4, annotation = indMA, main = "Microarra
y Datasets")
```

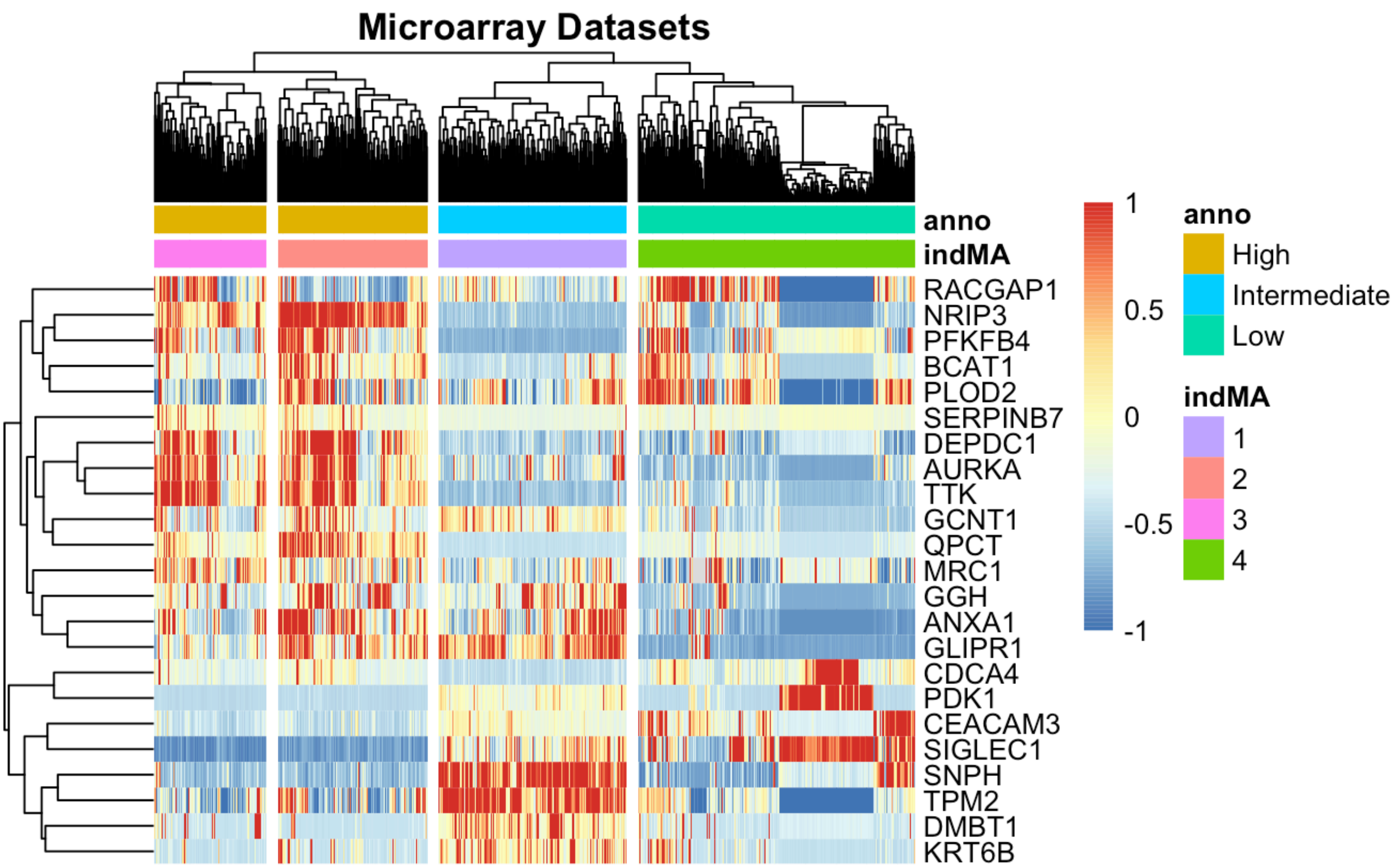

Then, we applied Kaplan-Meier curve to elucidate the prognostic value of 26 immune gene signature classification in the pooled cohort of CCA patients (GSE107943 and TCGA-CHOL)

Hide

```
#Annotate Samples with Cluster Group name
indGSE$samples <- rownames(indGSE)
indGSE$anno <- "High"
indGSE$anno[indGSE$indGSE == 1] <- "Low"

indTCGA$samples <- rownames(indTCGA)
indTCGA$anno <- "High"
indTCGA$anno[indTCGA$indTCGA == 2] <- "Low"
annotation <- rbind(indGSE[,2:3], indTCGA[,2:3])
#Plot Kaplain-Meier Curve using the Cluster annotation as a factor
GSETCGA <- rbind(GSEsurvgenes[,1:5], TCGAsurvgenes[,1:5])
GSETCGA <- merge(GSETCGA, annotation, by.x = "Sample", by.y = "samples", all.y = T
RUE)
km_fit <- survfit(Surv(OS, Death) ~ anno, data=GSETCGA)
ggsurv <- ggsurvplot(km_fit, data = GSETCGA,  risk.table = TRUE, cumevents = TRUE,
pval = TRUE, pval.method = TRUE, risk.table.col = "strata",
                    legend.labs=c("High", "Low"), palette = c("tomato", "cornflowe
rblue"))
ggsurv
```

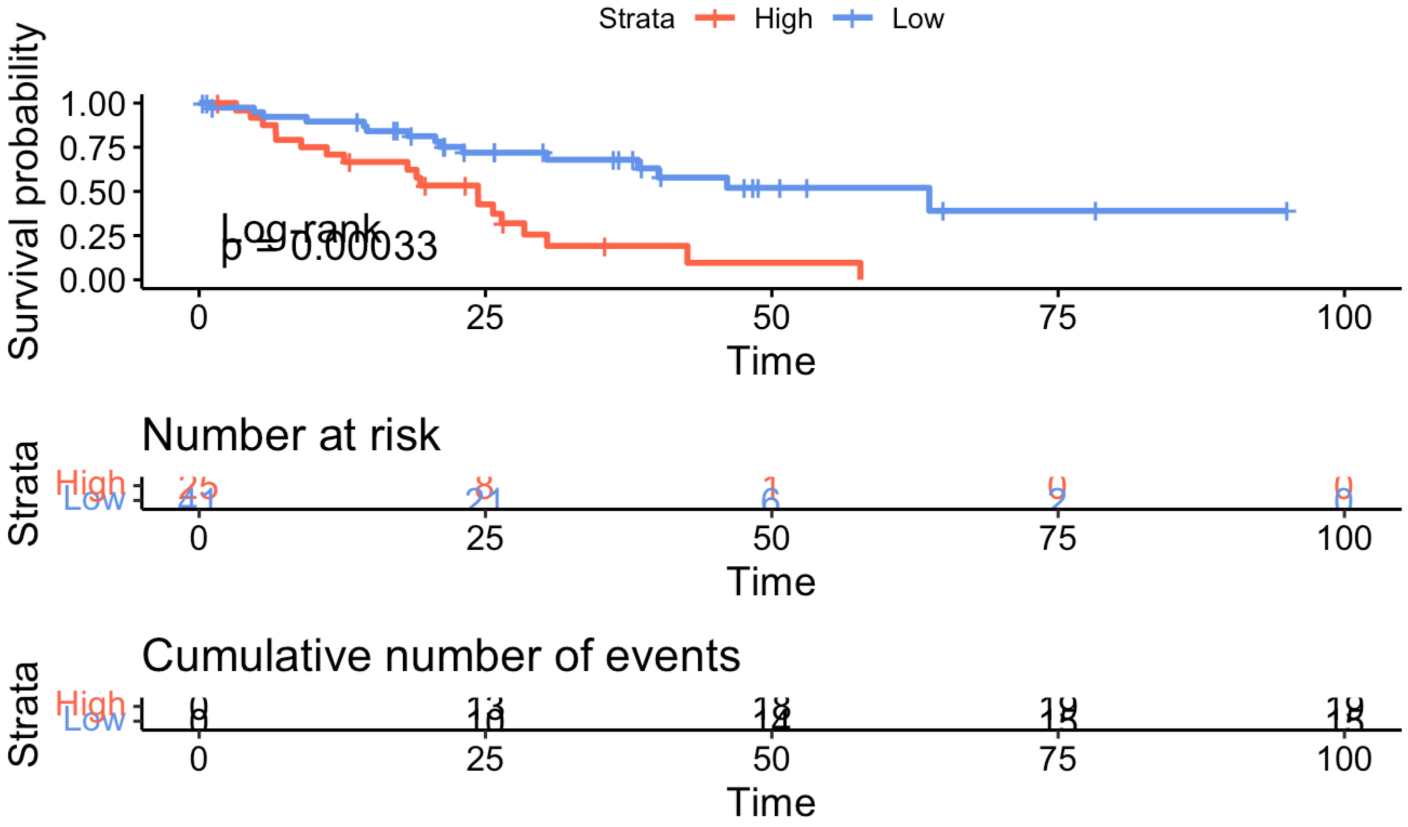

We have identified low expressing and high expressing samples in each cohort. We would now like to assess the transcriptomic differences between these two groups, hence we will conduct Differential Gene Expression Analysis.

Hide

```
#Load Libraries and Pre-process Data
library(DESeq2)
library(EnhancedVolcano)
library(pheatmap)
library(dplyr)
library(tibble)

#Data Preprocessing
GSEds <- column_to_rownames(GSEds, "Genes")
counts.m <- as.matrix(GSEds)
LHgroup <- indGSE
dds <- DESeqDataSetFromMatrix(countData = round(counts.m),
                              colData = LHgroup,
                             design = ~ anno)
keep <- rowSums(counts(dds)) >= 10
summary(keep)
dds <- dds[keep,]
#Differential Gene Expression Analysis
dds <- DESeq(dds)
res <- results(dds)
res
resOrdered <- res[order(res$pvalue),]
summary(res)
sum(res$padj < 0.1, na.rm=TRUE)
res1 <- as.data.frame(res)
res1 <- filter(res1, res1$padj < 0.05, na.rm=TRUE)
write.csv(as.data.frame(resOrdered),
          file="~/Desktop/Datasets/GSEimmune2DESeq2_HighLow.csv")
resSig <- subset(resOrdered, padj < 0.05)
resSig
write.csv(as.data.frame(resSig), file = "~/Desktop/Datasets/TableS5.csv")
```

Hide

```
#Plot PCA - GSE107943
rld <- rlog(dds, blind=TRUE)
```

```
rlog() may take a few minutes with 30 or more samples,
vst() is a much faster transformation
```

Hide

```
p <- plotPCA(rld, intgroup="anno")
p + scale_colour_manual(values = c("Low" = "cornflowerblue", "High" = "tomato"))
```

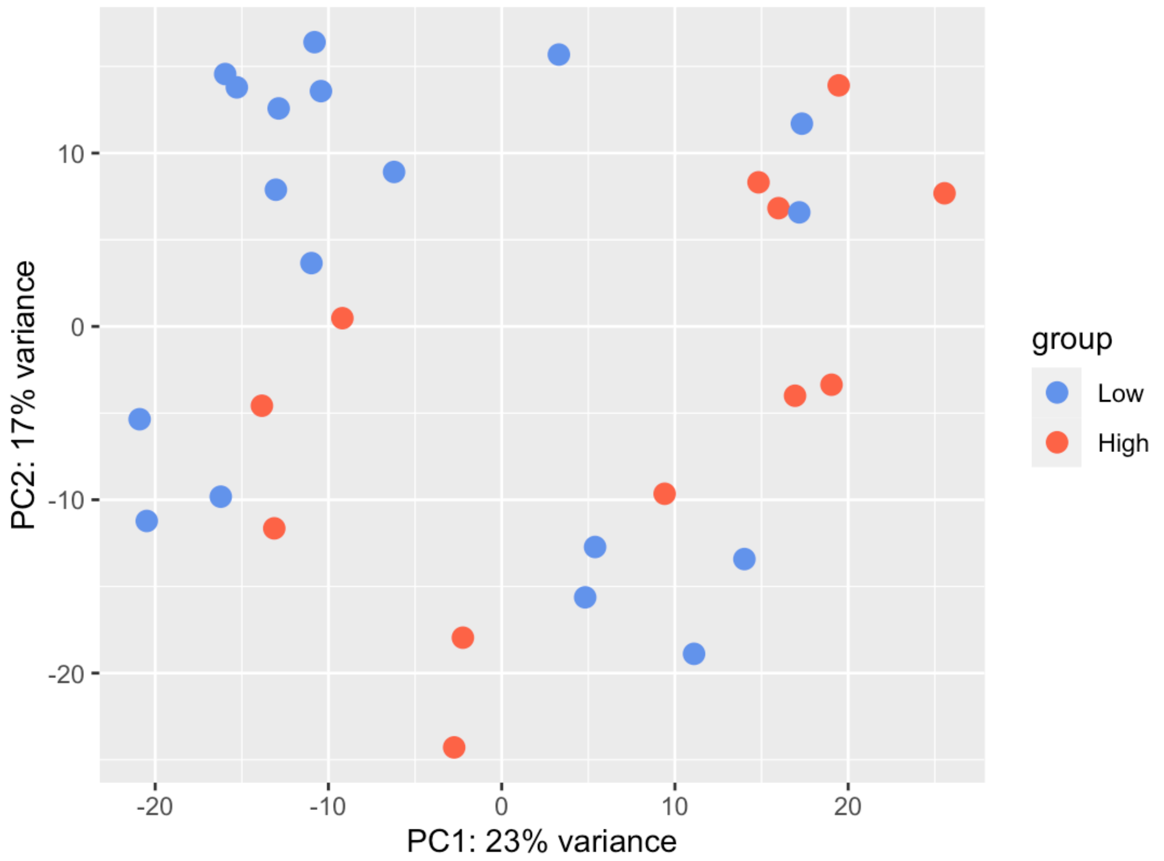

Hide

```
#Plot Volcano of Differentially Expressed Genes - GSE107943
EnhancedVolcano(res,
                lab = rownames(res),
                x = "log2FoldChange",
                y = "padj",
                ylim = c(0,4),
                title = "GSE107943",
                FCcutoff = log2(2),
                pCutoff = 0.05,
                legendPosition = "none",
                xlim = c(-5,5))
```

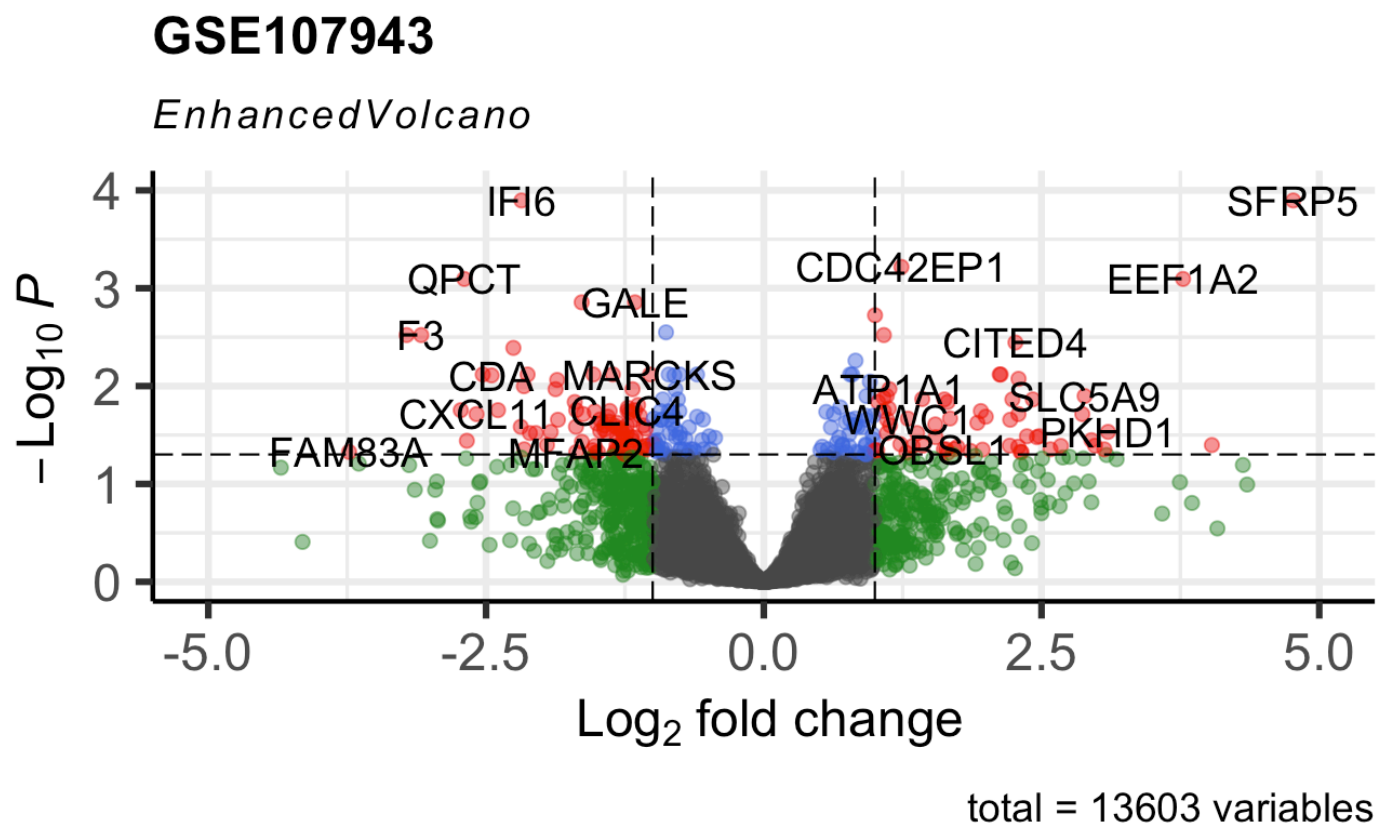

Hide

```
#Plot Heatmap of Differentially Expressed Genes - GSE107943
hmapx = subset(counts.m, rownames(counts.m) %in% rownames(resSig))
anno = LHgroup[,2:3]
rownames(anno) = NULL
anno = column_to_rownames(anno, "samples")
hmapx = hmapx[,-1]
pheatmap(hmapx, breaks = seq(-1.5, 1.5, length.out = 101), scale = "row",
         clustering_method = "complete", fontsize_row = 7, annotation_col = anno,
annotation_colors = list(anno = c(Low = "cornflowerblue", High = "tomato")),
         main = "GSE107943", border_color = NA, clustering_distance_cols = "canber
ra", annotation_names_col = F)
```

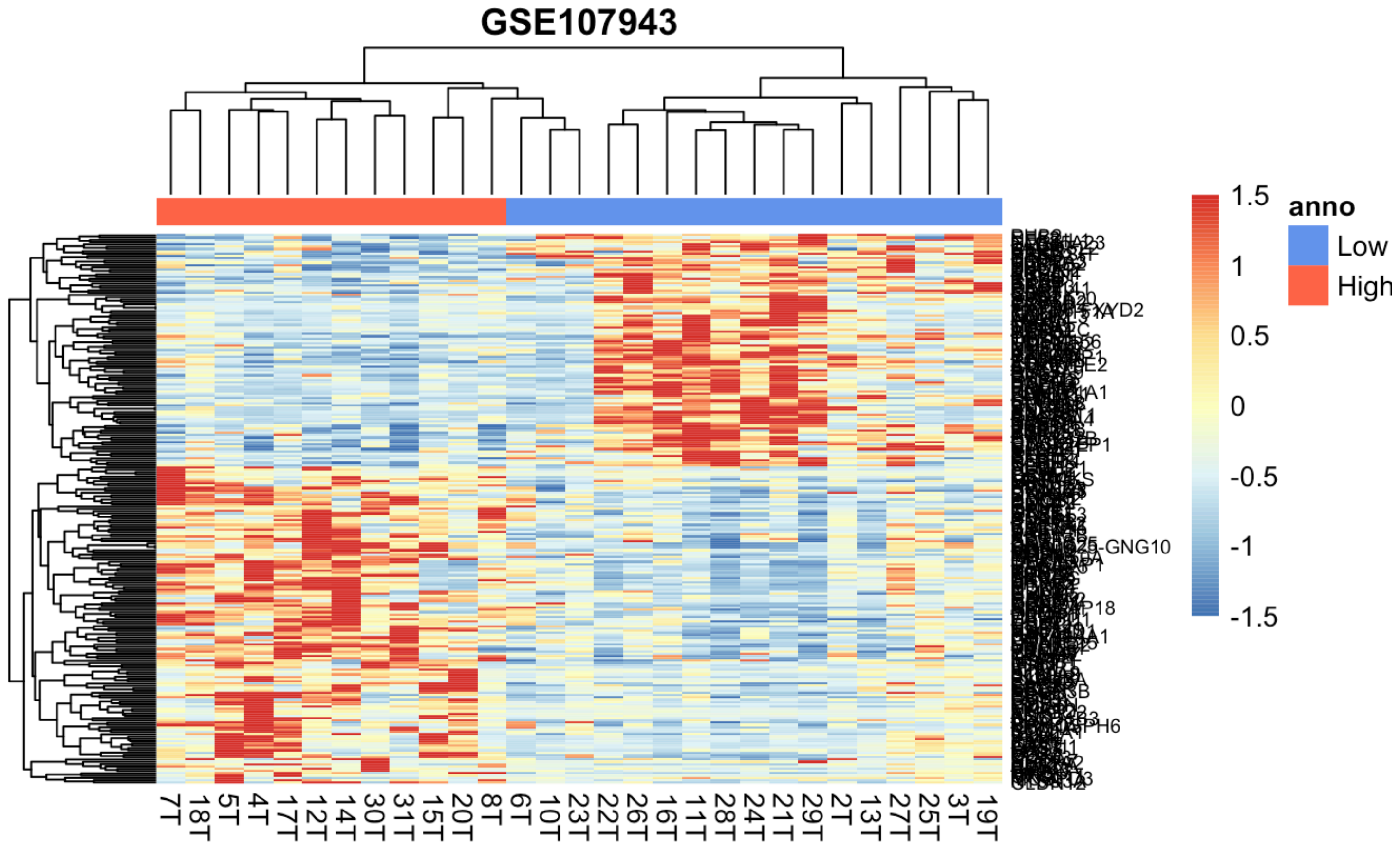

Now we will repeat this analysis for the TCGA-CHOL cohort

Hide

```
library(dplyr)
library(tibble)
library(tidyr)
#Repeat analysis in TCGA-CHOL cohort
#Data Pre-Processing
TCGAds = column_to_rownames(TCGAds, "Hugo_Symbol")
counts.m = as.matrix(TCGAds)
LHgroup = indTCGA
dds <- DESeqDataSetFromMatrix(countData = round(counts.m),
                              colData = LHgroup,
                              design = ~ anno)
keep <- rowSums(counts(dds)) >= 10
summary(keep)
dds <- dds[keep,]
#Differential Gene Expression Analysis
dds <- DESeq(dds)
res <- results(dds)
res
resOrdered <- res[order(res$pvalue),]
summary(res)
sum(res$padj < 0.1, na.rm=TRUE)
res1 = as.data.frame(res)
res1 = filter(res1, res1$padj < 0.05, na.rm=TRUE)
write.csv(as.data.frame(resOrdered),
          file="~/Desktop/Datasets/TCGAimmune2DESeq2_HighLow.csv")
resSig <- subset(resOrdered, padj < 0.05)
resSig
write.csv(as.data.frame(resSig), file = "~/Desktop/Datasets/TableS6.csv")
```

Hide

```
#Plot PCA - TCGA-CHOL
rld <- rlog(dds, blind=TRUE)
```

```
rlog() may take a few minutes with 30 or more samples,
vst() is a much faster transformation
```

Hide

```
p = plotPCA(rld, intgroup="anno")
p + scale_colour_manual(values = c("Low" = "cornflowerblue", "High" = "tomato"))
```

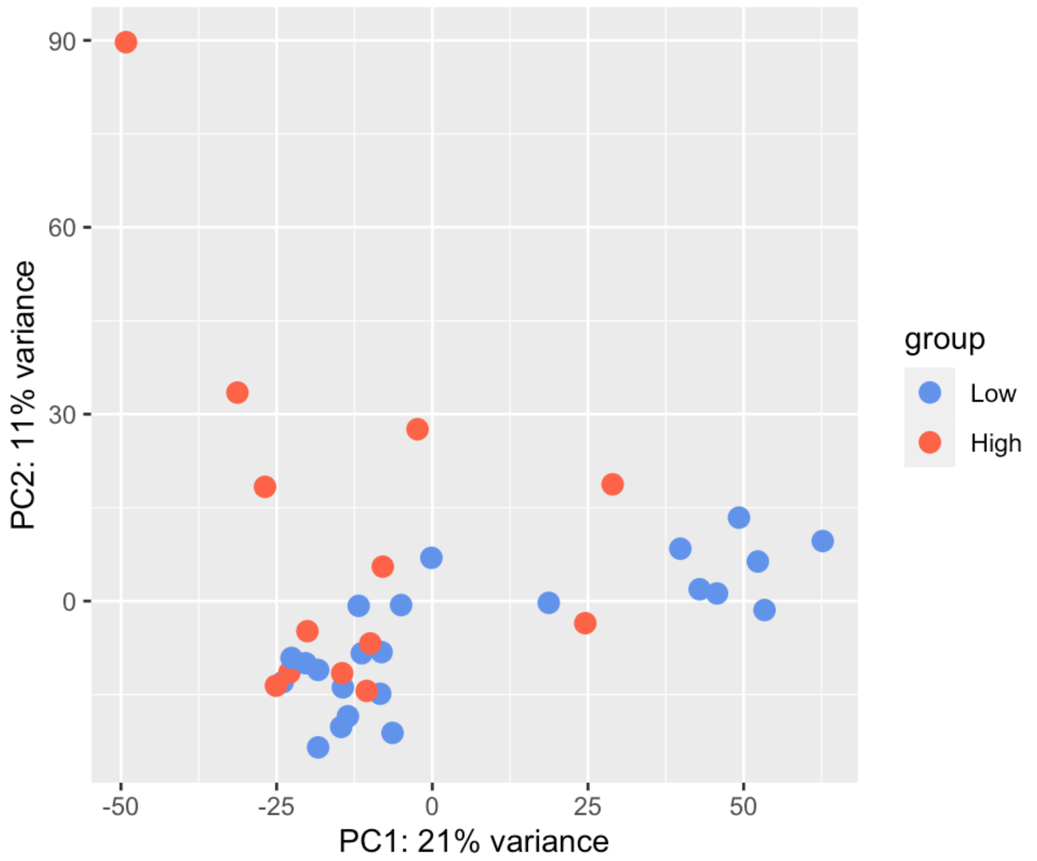

Hide

```
#Plot Volcano of Differentially Expressed Genes
EnhancedVolcano(res,
                lab = rownames(res),
                x = "log2FoldChange",
                y = "padj",
                ylim = c(0,4),
                title = "TCGA-CHOL",
                FCcutoff = log2(2),
                pCutoff = 0.05,
                legendPosition = "none",
                xlim = c(-5,5))
```

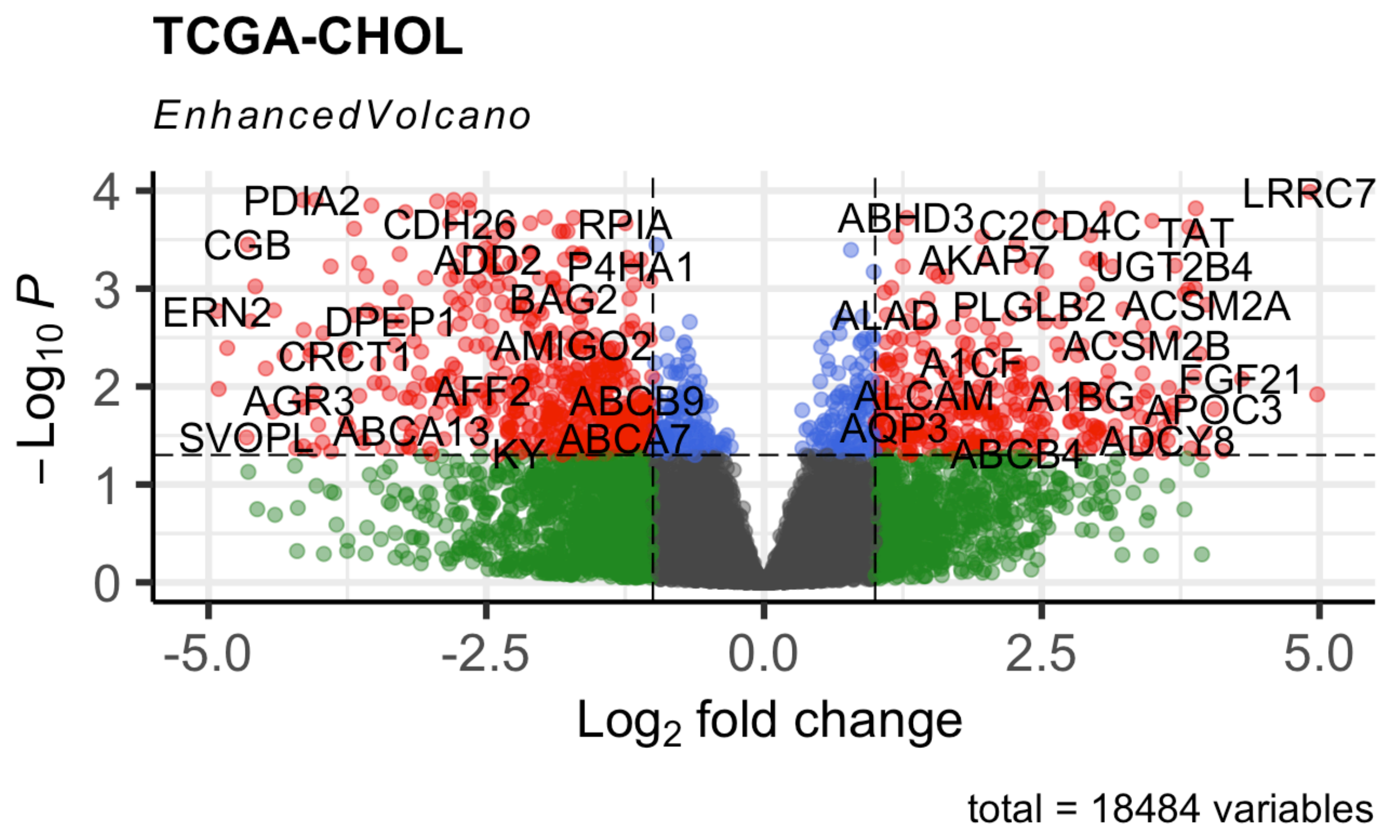

Hide

```
#Plot Heatmap of Differentially expressed genes
hmapx = subset(counts.m, rownames(counts.m) %in% rownames(resSig))
anno = LHgroup[,2:3]
rownames(anno) = NULL
anno = column_to_rownames(anno, "samples")
pheatmap(hmapx, breaks = seq(-1.5, 1.5, length.out = 101), show_colnames = T, scal
e = "row",
         clustering_method = "complete", fontsize_row = 7, annotation_col = anno,
annotation_colors = list(anno = c(Low = "cornflowerblue", High = "tomato")), clust
ering_distance_cols = "correlation",
         main = "TCGA-CHOL", border_color = NA, annotation_names_col = F)
```

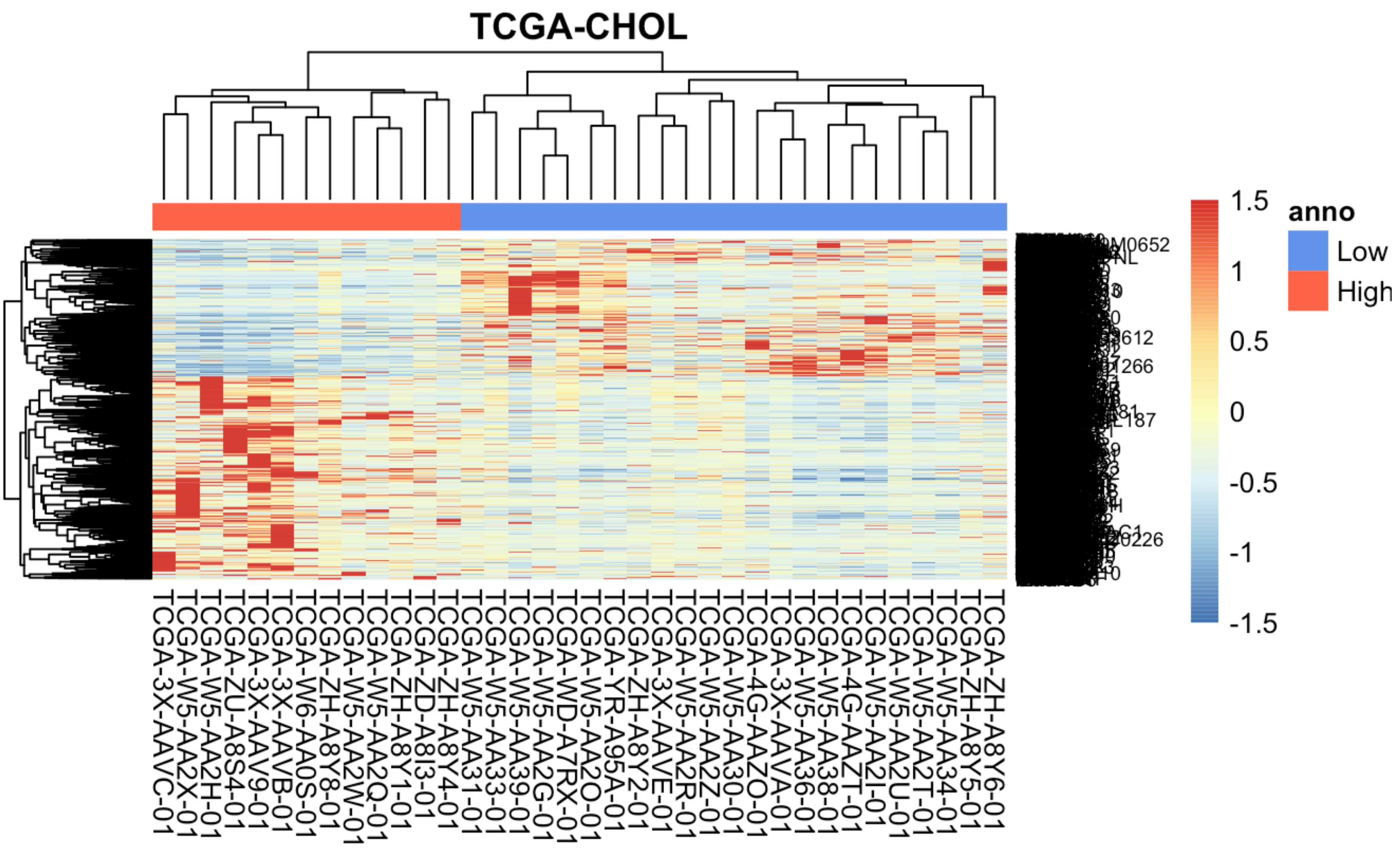

Hide

```
# Microarray DGE Validation
df <- as.data.frame(Microarrayds)
df = column_to_rownames(df, "GeneID")
counts <- as.matrix(df)
View(counts)

sample_id <- read_csv("~/Desktop/Datasets/MAhclustidentifiers.csv")
```

```
New names:
* `` -> ...1
```

```
Rows: 704 Columns: 3
── Column specification ────────────────────────────────────────────────────
────────
Delimiter: ","
chr (2): ...1, anno
dbl (1): indMA

ℹ Use `spec()` to retrieve the full column specification for this data.
ℹ Specify the column types or set `show_col_types = FALSE` to quiet this message.
```

Hide

```
View(sample_id)
sample_id = filter(sample_id, sample_id$anno == "Low"| sample_id$anno == "High")
colnames(sample_id)[1] = "samples"
sample_id = column_to_rownames(sample_id, "samples")
counts = t(counts)
counts = subset(counts, rownames(counts) %in% rownames(sample_id))
counts = t(counts)
genelist <- rownames(counts)
genes = genelist
group <- sample_id$anno
counts.m<-as.matrix(counts)
is.numeric(counts.m)
```

```
[1] TRUE
```

Hide

```
#Remove genes that are 0
n <- which(is.na(counts.m))
counts.m[n]<-0

#Transformation from raw  scale -->log2
counts.L = log2(counts.m + 1)
x <- DGEList(counts= counts.L, genes = genes, group = group, samples = sample_id$X
1)
group <- as.factor(group)
x$samples$group <- group
x$genes <- genes
x <- calcNormFactors(x, method = "TMM")

#plotMDS for unsupervised clustering
par(mfrow=c(1,2))
group <- as.factor(group)
col.group <- group
levels(col.group) <-  c("tomato", "cornflowerblue")
col.group <- as.character(col.group)
plotMDS(x, labels=group, col=col.group)
```

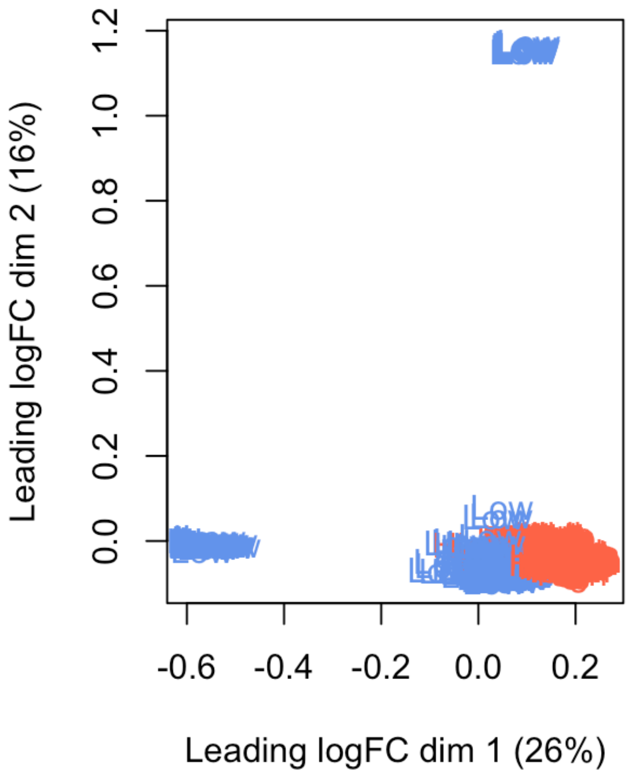

Hide

```
#title(main="Sample groups")
```

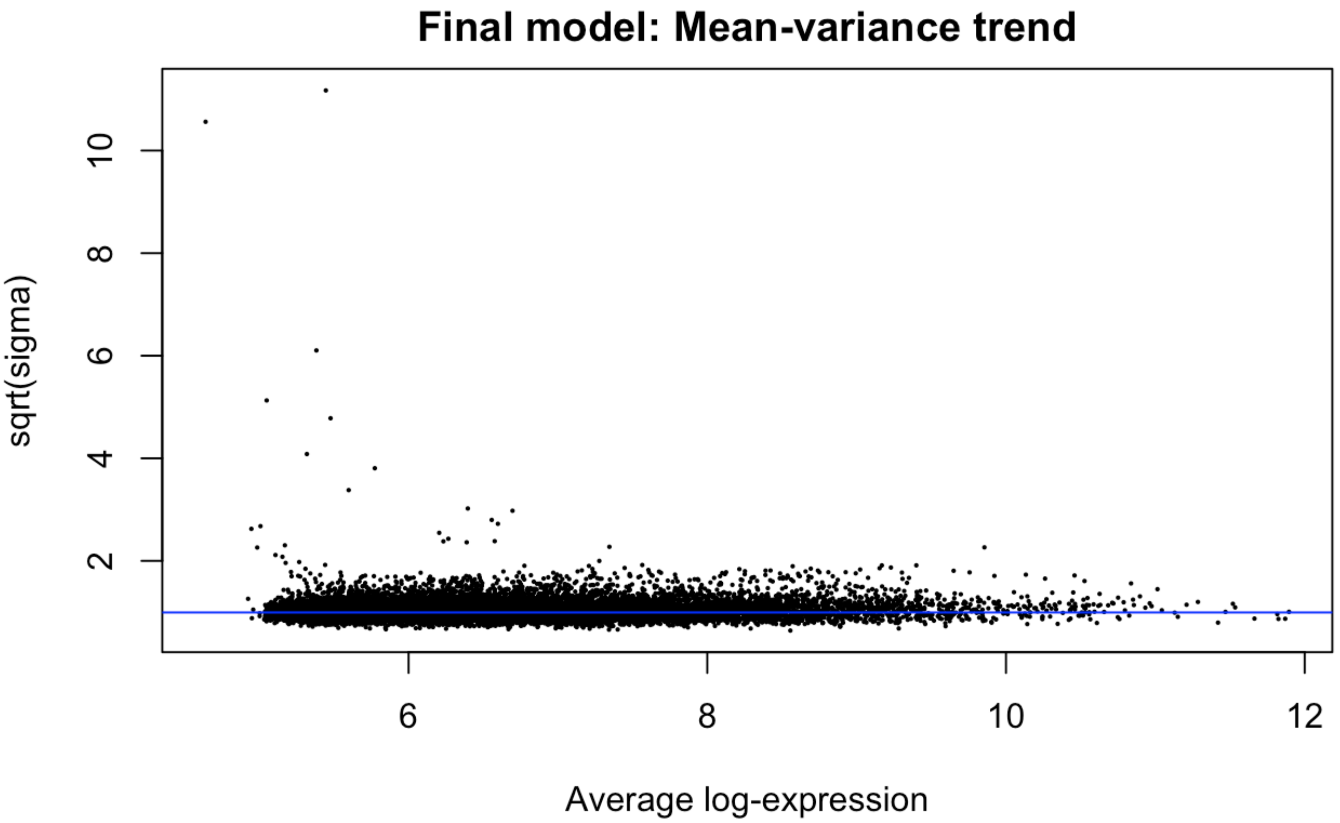

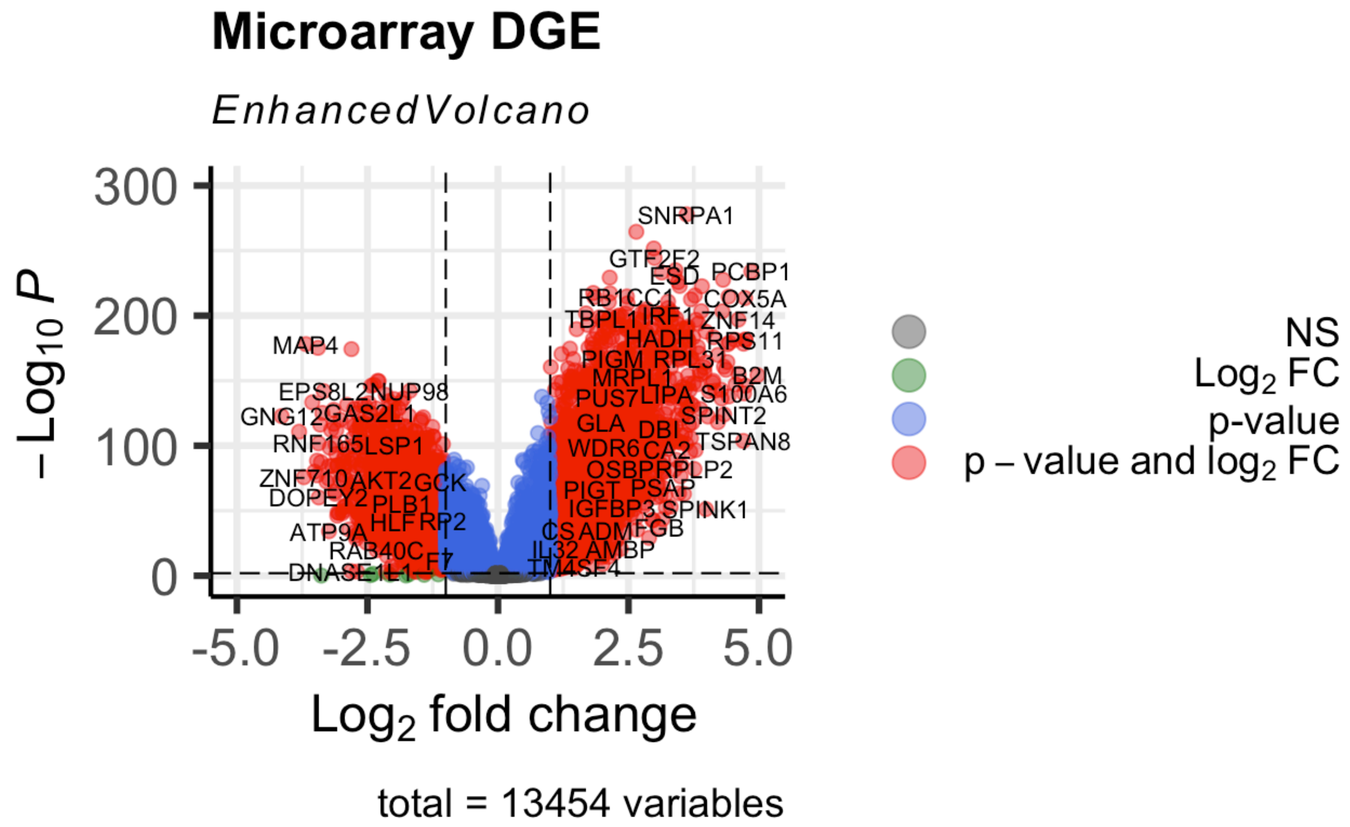

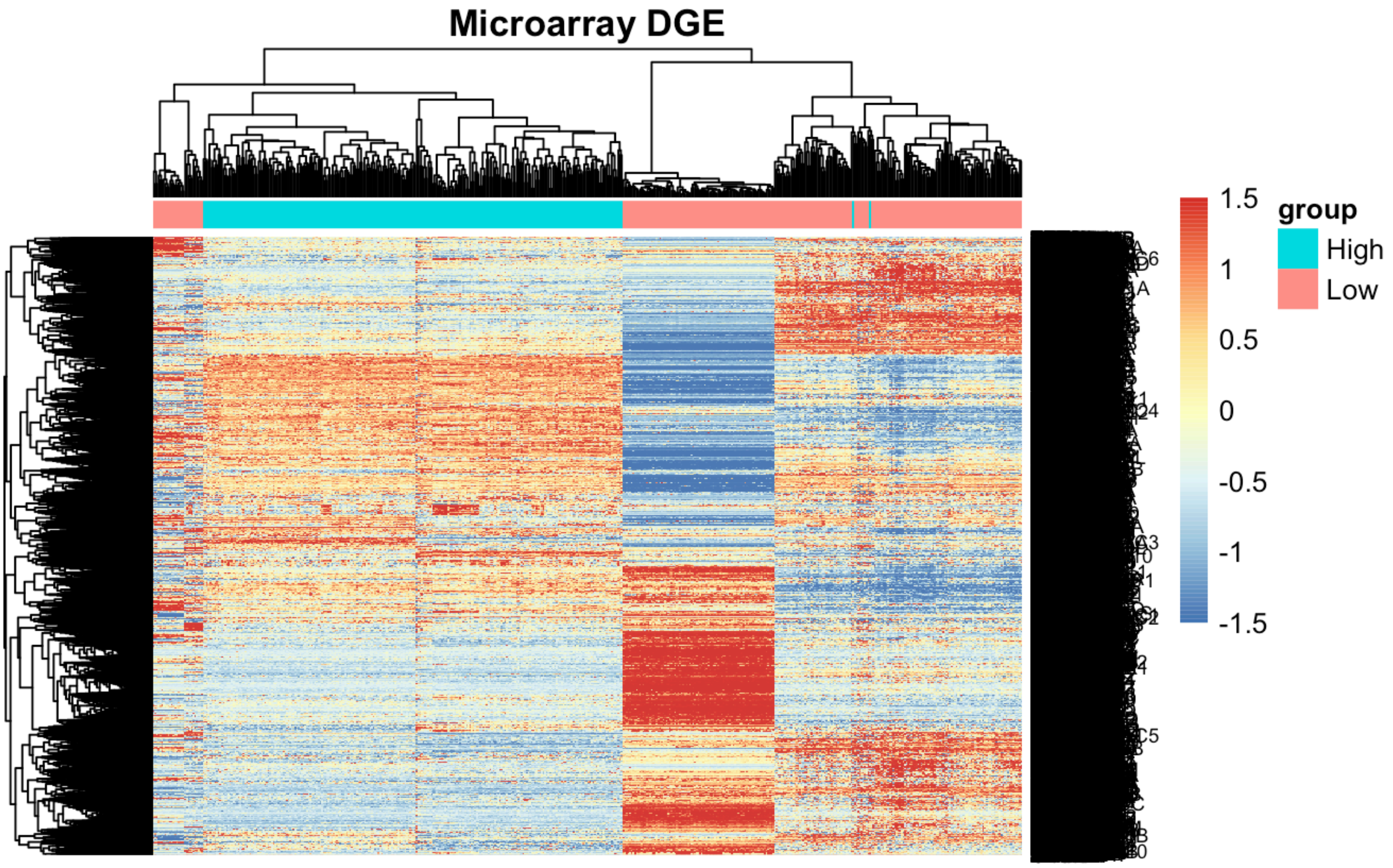

The differentially expressed gene list has been used as an input in iLINCS and EnrichR to obtain pathway enrichments and positively correlated connected perturbagens. These are presented in Table S6, S8, S10, S11 and S12. They have been visualized using the following script.

Hide

```
#Pathway Enrichment Plot
library(ggplot2)
Enrichment_GSE <- read_csv("~/Desktop/Datasets/Enrichment_GSE.csv")
```

```
Rows: 692 Columns: 10
── Column specification ────────────────────────────────────────────────────────
Delimiter: ","
chr (3): Term, Overlap, Database
dbl (7): P-value, Adjusted P-value, Old P-value, Old Adjusted P-value, Odds Ratio, C...

ℹ Use `spec()` to retrieve the full column specification for this data.
ℹ Specify the column types or set `show_col_types = FALSE` to quiet this message.
```

Hide

```
Enrichment_TCGA <- read_csv("~/Desktop/Datasets/Enrichment_TCGA.csv")
```

```
Rows: 488 Columns: 10
── Column specification ────────────────────────────────────────────────────────
Delimiter: ","
chr (3): Term, Overlap, Database
dbl (7): P-value, Adjusted P-value, Old P-value, Old Adjusted P-value, Odds Ratio, C...

ℹ Use `spec()` to retrieve the full column specification for this data.
ℹ Specify the column types or set `show_col_types = FALSE` to quiet this message.
```

Hide

```
Enrichment_MA <- read_csv("~/Desktop/Datasets/Enrichment_MA.csv")
```

```
Rows: 453 Columns: 10
── Column specification ────────────────────────────────────────────────────────
Delimiter: ","
chr (3): Term, Overlap, Database
dbl (7): P-value, Adjusted P-value, Old P-value, Old Adjusted P-value, Odds Ratio, C...

ℹ Use `spec()` to retrieve the full column specification for this data.
ℹ Specify the column types or set `show_col_types = FALSE` to quiet this message.
```

Hide

```
ds <- data.frame(cbind(Enrichment_GSE$`Term`, Enrichment_GSE$`DEGs`, Enrichment_GS
E$`Combined Score`, Enrichment_GSE$`Adjusted P-value`, Enrichment_GSE$Database))
colnames(ds) <- c("Pathways", "DEGs", "Combined Score", "Adjusted P.val", "Databas
e")
Enrichment_GSE <- as.data.frame(Enrichment_GSE)
ds[,c(2:4)] <- sapply(ds[,c(2:4)], as.numeric)
pathway_GSE = ggplot(ds ,aes(x= `Combined Score`,
                    y= Pathways, size = DEGs))+geom_point(aes(col= `Adjusted P.val`)
, alpha=0.8)+scale_colour_gradient(low = "red", high = "blue")+scale_y_discrete(la
bels = function(x) str_wrap(x, width = 10))+facet_grid(~Database)+xlim(0, 500)
pathway_GSE
```

```
Warning: Removed 2 rows containing missing values (geom_point).
```

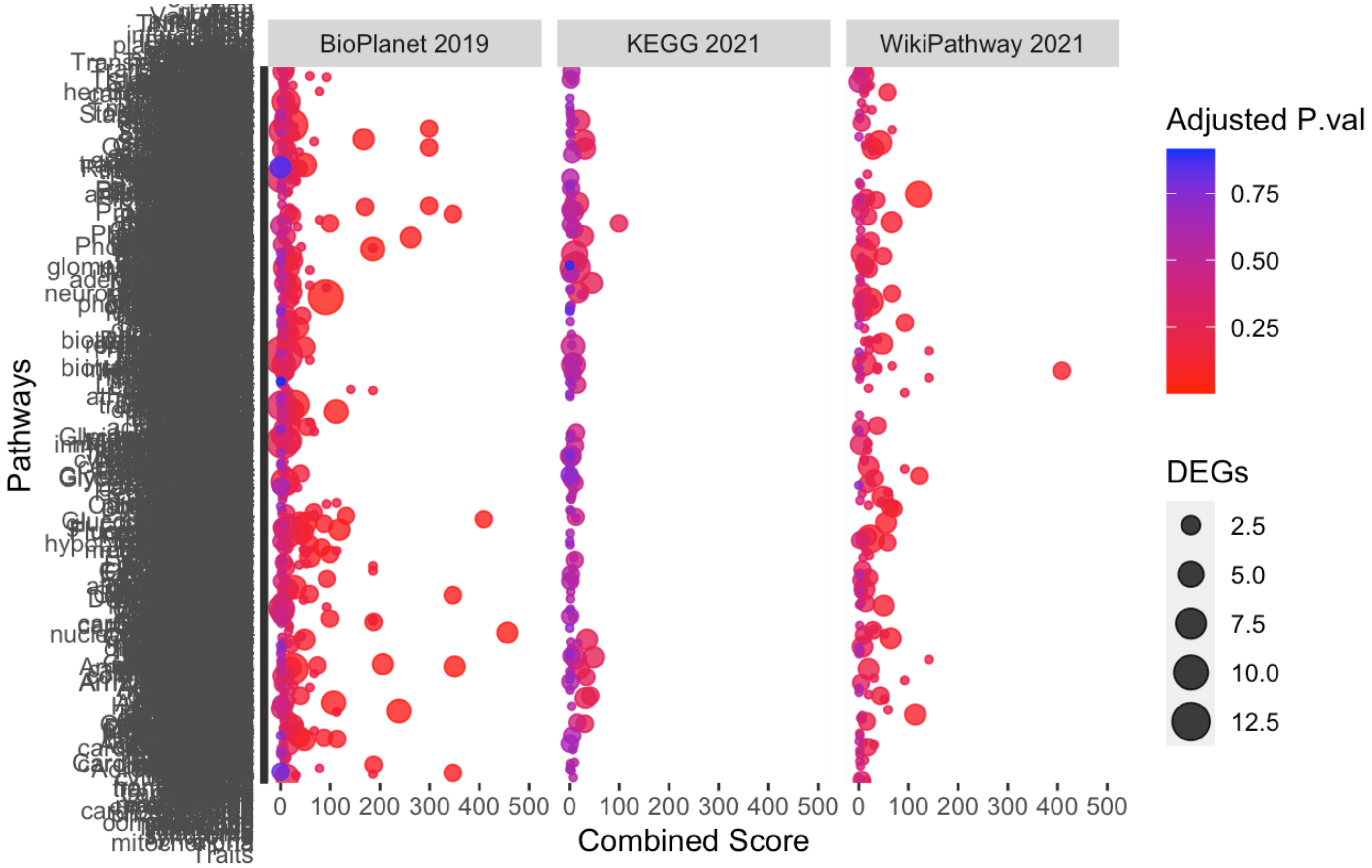

Hide

```
ds <- data.frame(cbind(Enrichment_TCGA$`Term`, Enrichment_TCGA$`DEGs`, Enrichment_TCGA$`Combined Score`, Enrichment_TCGA$`Adjusted P-value`, Enrichment_TCGA$Database))
colnames(ds) <- c("Pathways", "DEGs", "Combined Score", "Adjusted P.val", "Database")
Enrichment_TCGA <- as.data.frame(Enrichment_TCGA)
ds[,c(2:4)] <- sapply(ds[,c(2:4)], as.numeric)

pathway_TCGA <- ggplot(ds ,aes(x= `Combined Score`,
                   y= Pathways, size = DEGs))+geom_point(aes(col= `Adjusted P.val`), alpha=0.8)+scale_colour_gradient(low = "red", high = "blue")+scale_y_discrete(labels = function(x) str_wrap(x, width = 10))+facet_grid(~Database)+xlim(0, 500)
pathway_TCGA
```

```
Warning: Removed 1 rows containing missing values (geom_point).
```

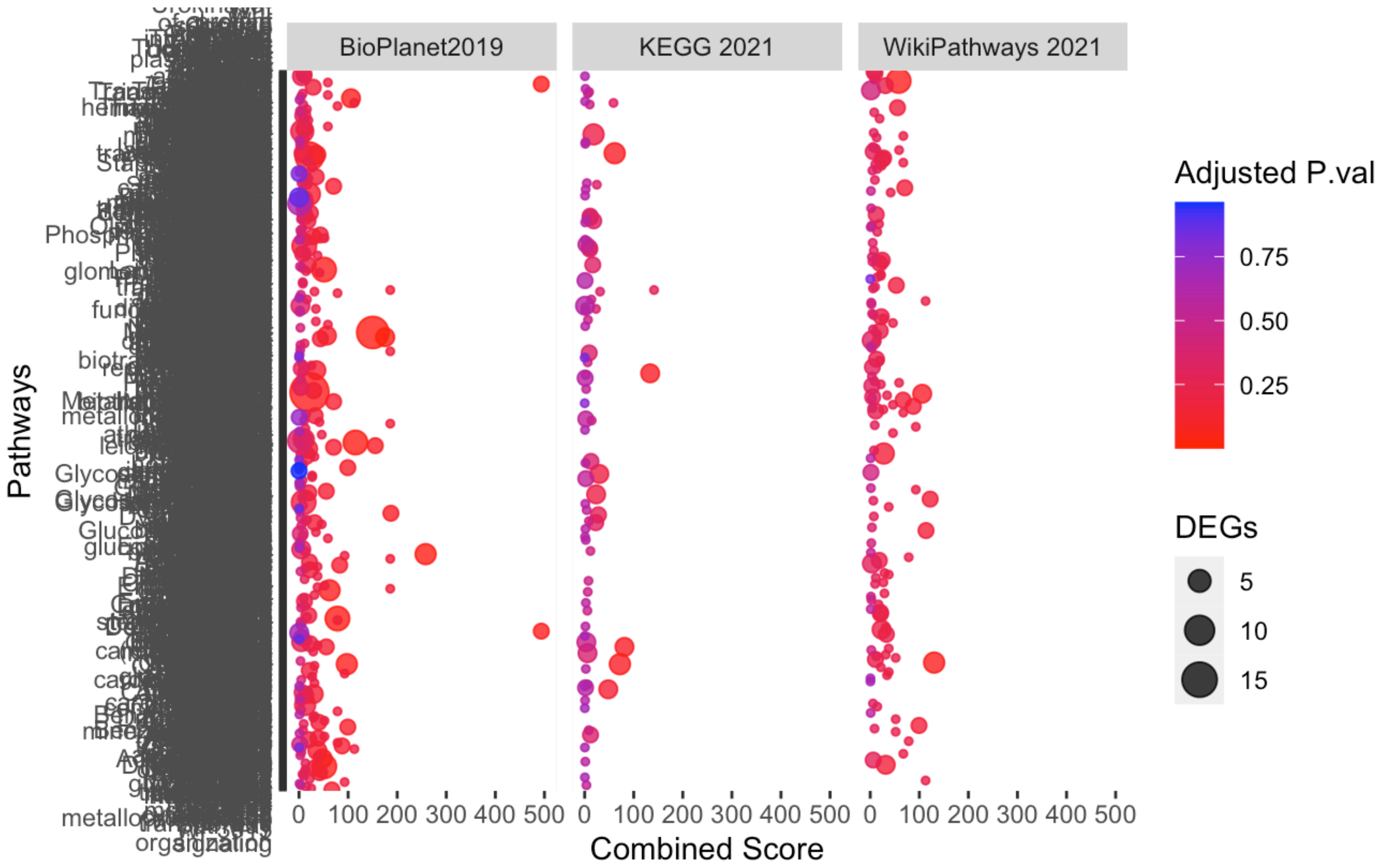

Hide

```
ds = data.frame(cbind(Enrichment_MA$`Term`, Enrichment_MA$`DEGs`, Enrichment_MA$`C
ombined Score`, Enrichment_MA$`Adjusted P-value`, Enrichment_MA$Database))
colnames(ds) <- c("Pathways", "DEGs", "Combined Score", "Adjusted P.val", "Databas
e")
Enrichment_MA <- as.data.frame(Enrichment_MA)
ds[,c(2:4)] <- sapply(ds[,c(2:4)], as.numeric)
pathway_MA <- ggplot(ds ,aes(x= `Combined Score`,
                  y= Pathways, size = DEGs))+geom_point(aes(col= `Adjusted P.val`)
, alpha=0.8)+scale_colour_gradient(low = "red", high = "blue")+scale_y_discrete(la
bels = function(x) str_wrap(x, width = 10))+facet_grid(~Database)+xlim(0, 500)
pathway_MA
```

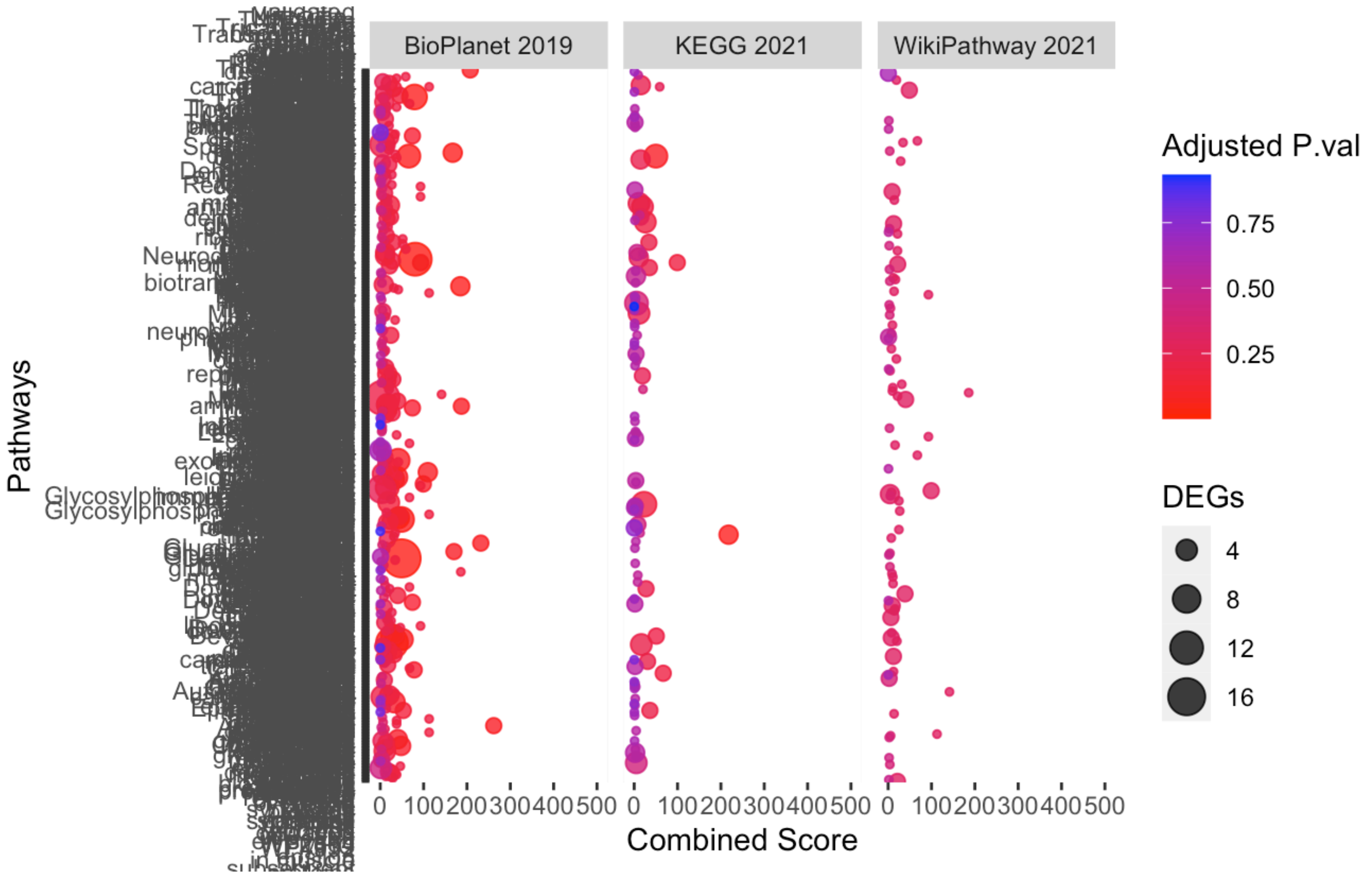

Hide

```
#Connected Perturbagens Plot
GSEdrugs <- read_tsv("~/Desktop/Datasets/GSEdrugs.txt")
```

```
Rows: 312 Columns: 7
── Column specification ────────────────────────────────────────────────────────
────
Delimiter: "\t"
chr (4): PerturbagenId, Perturbagen, GeneTargets, Correlation
dbl (3): NoOfSignatures, pValue, zScore

ℹ Use `spec()` to retrieve the full column specification for this data.
ℹ Specify the column types or set `show_col_types = FALSE` to quiet this message.
```

Hide

```
TCGAdrugs <- read_tsv("~/Desktop/Datasets/TCGAdrugs.txt")
```

```
Rows: 1604 Columns: 7
── Column specification ────────────────────────────────────────────────────────
Delimiter: "\t"
chr (4): PerturbagenId, Perturbagen, GeneTargets, Correlation
dbl (3): NoOfSignatures, pValue, zScore

ℹ Use `spec()` to retrieve the full column specification for this data.
ℹ Specify the column types or set `show_col_types = FALSE` to quiet this message.
```

Hide

```
MAdrugs <- read_tsv("~/Desktop/Datasets/MAdrugs.txt")
```

```
Rows: 1780 Columns: 7
── Column specification ────────────────────────────────────────────────────────
Delimiter: "\t"
chr (4): PerturbagenId, Perturbagen, GeneTargets, Correlation
dbl (3): NoOfSignatures, pValue, zScore

ℹ Use `spec()` to retrieve the full column specification for this data.
ℹ Specify the column types or set `show_col_types = FALSE` to quiet this message.
```

Hide

```
GSEdrugs %>% arrange(desc(zScore)) %>% head(25) %>%ggplot(aes(reorder(Perturbagen,
zScore), zScore))+
  geom_col(aes(fill = pValue)) +
  scale_fill_gradient(low = "darkgoldenrod1",
                      high = "darkgoldenrod4") +
  scale_x_discrete(labels = function(x) str_wrap(x, width = 10))+
  coord_flip() +
  labs(x = "Connected Perturbagens", y = "Z score")
```

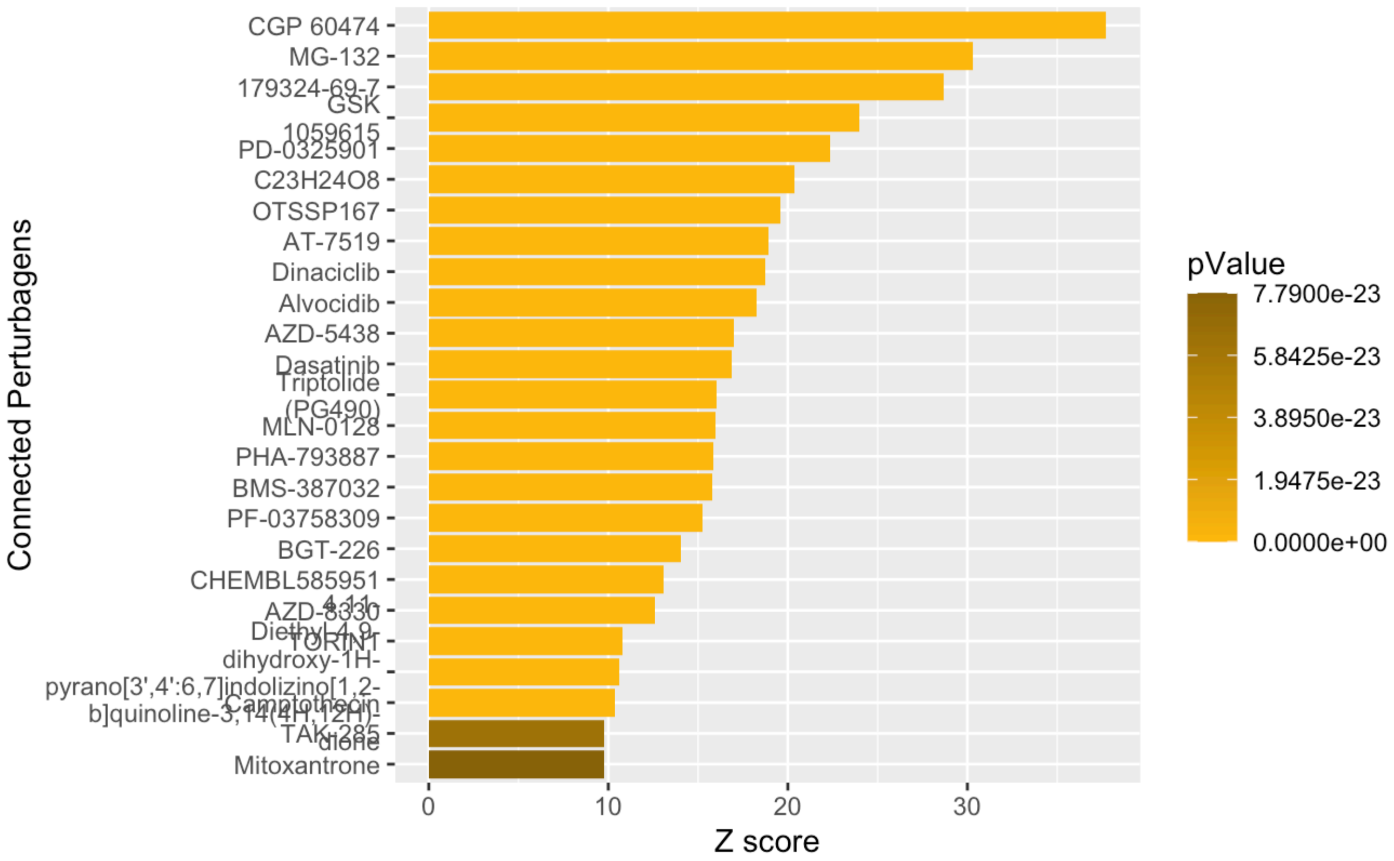

Hide

```
TCGAdrugs %>% arrange(desc(zScore)) %>% head(25) %>%ggplot(aes(reorder(Perturbagen
, zScore), zScore))+
  geom_col(aes(fill = pValue)) +
  scale_fill_gradient(low = "darkolivegreen2",
                      high = "darkolivegreen4") +
  coord_flip() +
  labs(x = "Connected Perturbagens", y = "Z score")
```

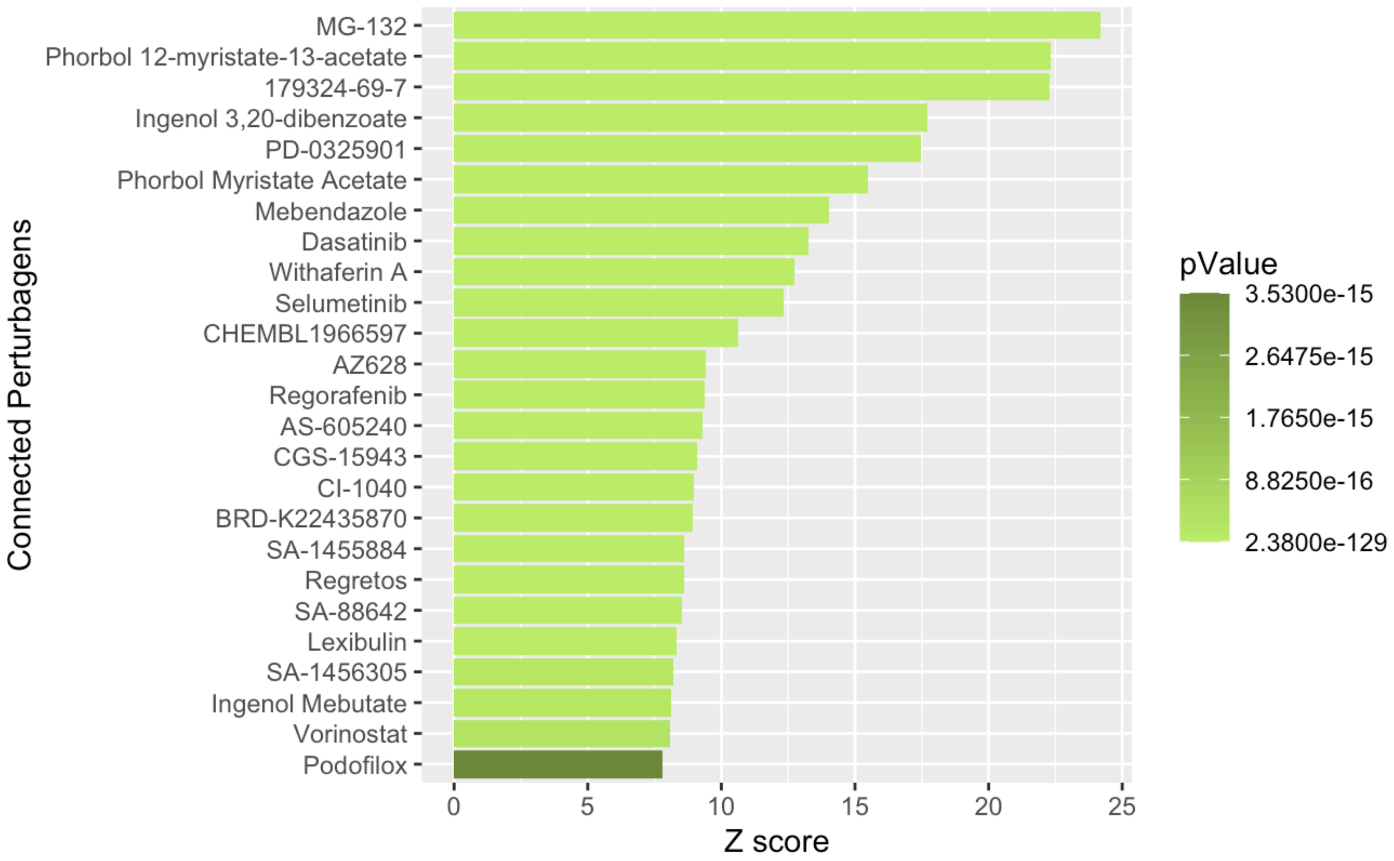

Hide

```
MAdrugs %>% arrange(desc(zScore)) %>% head(25) %>%ggplot(aes(reorder(Perturbagen,
zScore), zScore))+
  geom_col(aes(fill = pValue)) +
  scale_fill_gradient(low = "deepskyblue",
                      high = "deepskyblue4") +
  coord_flip() +
  labs(x = "Connected Perturbagens", y = "Z score")
```

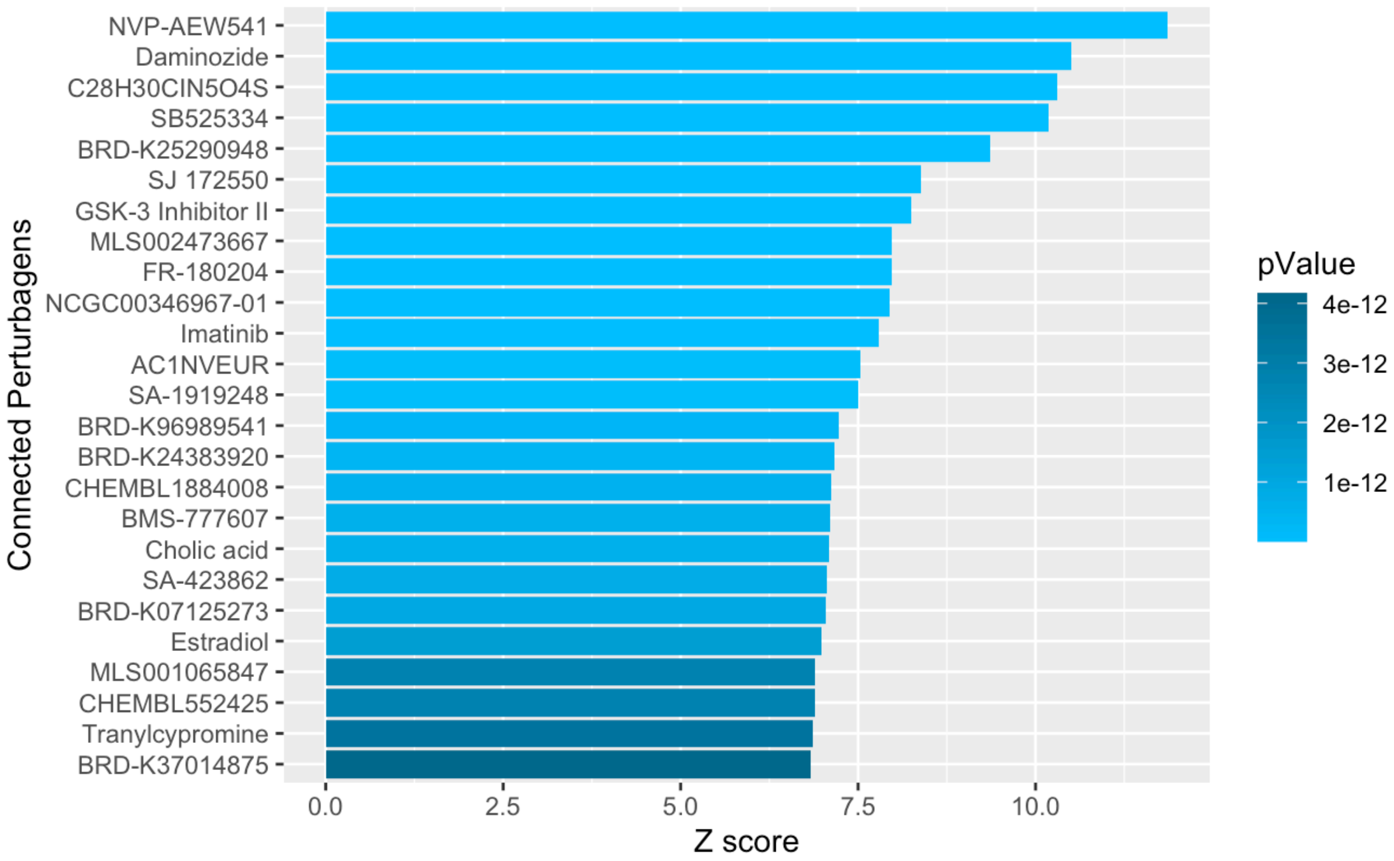
NVP-AEW541
Daminozide
C28H30CIN5O4S
SB525334
BRD-K25290948
SJ 172550
GSK-3 Inhibitor II
MLS002473667
FR-180204
NCGC00346967-01
Imatinib
AC1NVEUR
SA-1919248
BRD-K96989541
BRD-K24383920
CHEMBL1884008
BMS-777607
Cholic acid
SA-423862
BRD-K07125273
Estradiol
MLS001065847
CHEMBL552425
Tranylcypromine
BRD-K37014875
Connected Perturbagens
0.0
2.5
5.0
7.5
10.0
Z score
pValue
4e-12
3e-12
2e-12
1e-12
